# The framework of Systematic Assessment for Resilience (SAR): development and validation

**DOI:** 10.1186/s12909-023-04177-5

**Published:** 2023-04-05

**Authors:** Majed Mohammed Wadi, Muhamad Saiful Bahri Yusoff, Mohamed Hassan Taha, Sarra Shorbagi, Nik Ahmad Zuky Nik Lah, Ahmad Fuad Abdul Rahim

**Affiliations:** 1 Medical Education Department, College of Medicine, Qassim University, Buraydah, Saudi Arabia; 2 Medical Education Department, School of Medical Sciences, Universiti Sains Malaysia, Kota Bharu, Kelantan Malaysia; 3 College of Medicine and Center of Medical Education, University of Sharjah, Sharjah, United Arab Emirates; 4 Department of Family and Community Medicine and Behavioral Science, College of Medicine, University of Sharjah, Sharjah, United Arab Emirates; 5 Obstetrics and Gynecology Department, School of Medical Sciences, Universiti Sains Malaysia, Kota Bharu, Kelantan Malaysia

**Keywords:** Health professions education, Test anxiety, Assessment, Student well-being, Resilience

## Abstract

**Background:**

Burnout and depression among health professions education (HPE) students continue to rise, leading to unwanted effects that ultimately jeopardise optimal medical care and patient health. Promoting the resilience of medical students is one solution to this issue. Several interventions have been implemented to foster resilience, but they focus on aspects other than the primary cause: the assessment system. The purpose of this study is to develop a framework to promote resilience in assessment planning and practice.

**Methods:**

We followed the guidelines suggested by Whetten for constructing a theoretical model for framework development. There were four phases in the model development. In the first phase, different literature review methods were used, and additional students’ perspectives were collected through focus group discussions. Then, using the data, we constructed the theoretical model in the second phase. In the third phase, we validated the newly developed model and its related guidelines. Finally, we performed response process validation of the model with a group of medical teachers.

**Results:**

The developed systematic assessment resilience framework (SAR) promotes four constructs: self-control, management, engagement, and growth, through five phases of assessment: assessment experience, assessment direction, assessment preparation, examiner focus, and student reflection. Each phase contains a number of practical guidelines to promote resilience. We rigorously triangulated each approach with its theoretical foundations and evaluated it on the basis of its content and process. The model showed high levels of content and face validity.

**Conclusions:**

The SAR model offers a novel guideline for fostering resilience through assessment planning and practice. It includes a number of attainable and practical guidelines for enhancing resilience. In addition, it opens a new horizon for HPE students’ future use of this framework in the new normal condition (post COVID 19).

**Supplementary Information:**

The online version contains supplementary material available at 10.1186/s12909-023-04177-5.

## Background

The study of medicine is demanding and puts a significant strain on the mental and physical health of medical students, who perceive medical education as an anxiety-inducing and stressful field of study [1]. This perception has been mirrored by the high level of stress in medical students. Several systematic reviews and metanalyses [2–4], as well as local [5, 6] and multicenter studies [7, 8] have found a significant prevalence of stress among medical students ranging from 21 to 56%. Consequently, burnout and depression among medical students have increased [4]. Other negative effects of stress on medical students have also been reported, including poor clinical performance, poor decision-making, poor peer interaction, interpersonal conflict, academic dishonesty, and sleep problems [4, 9]. Stress has also been associated with suicide, alcoholism, and drug abuse [10–13]. These negative effects eventually jeopardise optimal medical care and impact patient health negatively [14, 15]. While medical students have identified a number of stressors, research indicates that examinations are the most frequently reported sources of stress [14, 16–21]. As a result, there is a growing body of research on how to improve the mental health of medical students and promote what is currently known as resilience.

Resilience is a psychological construct that refers to the characteristics needed to adapt to the dynamic changes of life and maintain mental well-being [22]. The topic of resilience is of interest in many disciplines, including developmental psychology, sociology, education and, in particular, health professions education (HPE) [23–27]. In psychology, resilience generally refers to the status of an individual who is adapting to significant adversity while maintaining good mental and physical well-being [28]. Alva [29] defined academically resilient students as those ‘who sustain high levels of achievement motivation and performance despite the presence of stressful events and conditions that place them at risk of doing poorly in school and ultimately dropping out of school’ [29]. Further, academically resilient students are able to maintain mental agility and continue growing and developing despite academic and life setbacks [30–33]. In a recent meta-analysis of resilience constructs across 21 resilience scales, Wadi et al. [34] identified four primary resilience characteristics: 1) control: maintaining composure and control in the face of stressful adversity; 2) involvement: being committed to overcoming adversity; 3) resourcefulness: using available resources for appropriate solutions to overcome adversity; and 4) growth: continuing to grow and rebounding stronger from adversity. These four constructs provide a solid foundation for the implementation of interventions fostering resilience.

Studies on HPE have shown that resilience is positively correlated with compassion, satisfaction, and patient care and negatively associated with burnout, secondary stress, anxiety, intolerance to ambiguity, and poor communication [35]. Numerous health institutions have implemented interventions based on these studies [15, 36, 37]. Common intervention guidelines include training workshops focussed on psychosocial skills, such as mindfulness, Stress Management and Resilience Training (SMART), and narrative and simulation training [38–41]. Although these interventions have been shown to have some positive effects [42], they lack a solid theoretical foundation and do not focus on assessment, the primary source of student stress. Resilience theory must be integrated with the assessment context to provide a solid basis to guide intervention strategies and maintain the quality of assessment. Interestingly, a recent paper explored the intersection between resilience and curricula. The authors presented a principle-based approach to curriculum design to foster resilience as an integral part of the curriculum in higher education [43]. Although this approach sheds light on the philosophical approach to building a curriculum to create resilient graduates, it does not address the exact link between assessment and students’ resilience. To the best of our knowledge, no study has incorporated resilience into the assessment process. Accordingly, the purpose of this study was to develop a framework to promote resilience in assessment planning and practice. In this way, assessment would serve as a source of resilience and promote the development of resilience-improving characteristics among students.

## Methods

**Fig. 1:**
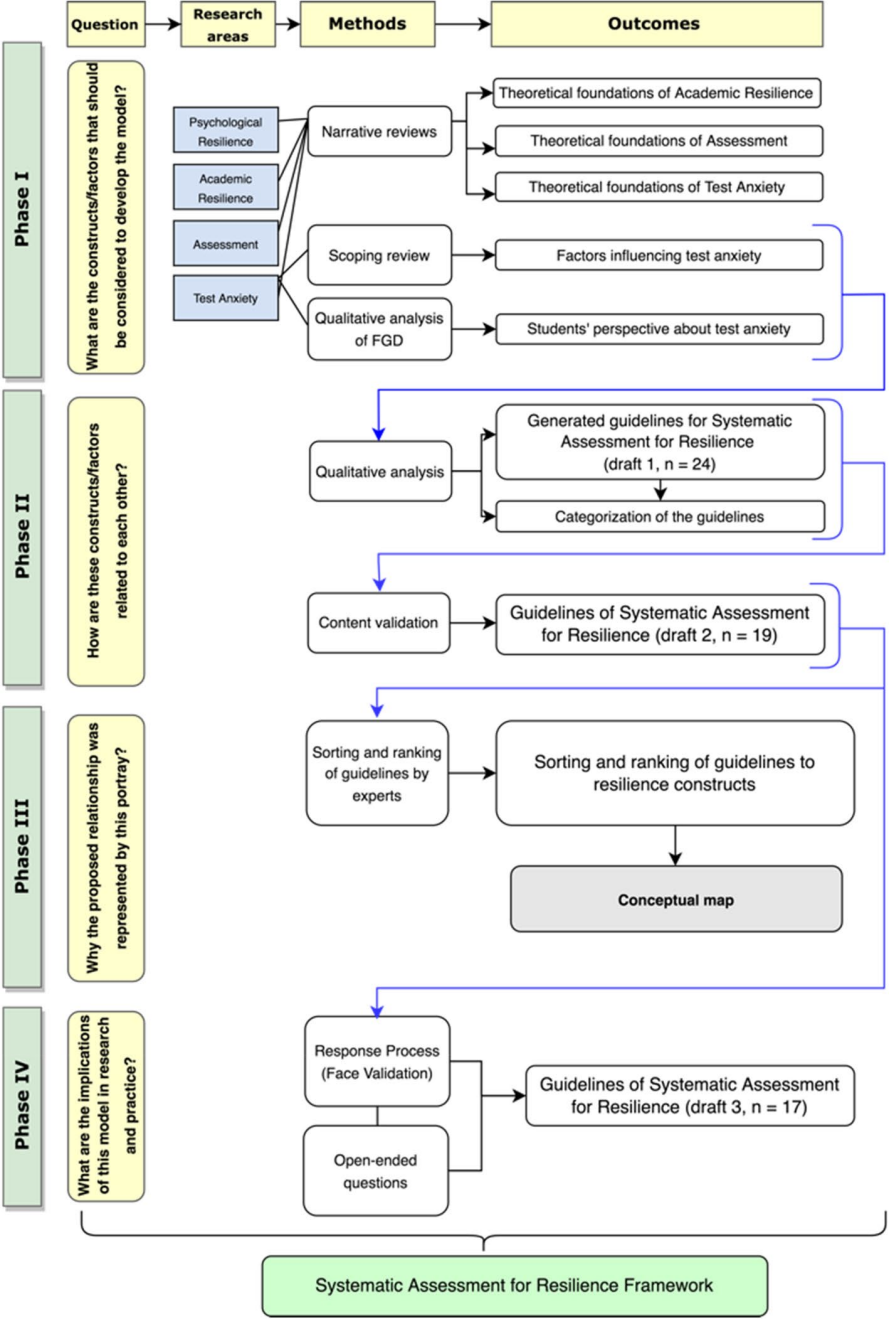
Flow chart summarizing study phases, questions, research areas, methods, and outcomes of each phase

The researchers followed the guidelines for developing a theoretical model proposed by Whetten [44]. These guidelines include four essential questions for model development: (1) What are the constructs/factors that should be considered to explain the model? (2) How are these constructs/factors related to each other? (3) Why is the proposed relationship represented by this portray? (4) What are the implications of this model for research and practice? Each question refers to a developmental phase of the theoretical model. Accordingly, the researchers developed the model in these four phases. This involved establishing the foundation (literature review and focus group discussion) and triangulating the findings [45] through a content validation and response process. Figure 1 presents a flow chart summarising the study phases, questions, research areas, methods, and outcomes of each phase.

### Phase I

#### Identifying resilience constructs

In this phase, we aimed to answer the following question: *‘What are the constructs/factors that should be considered in developing the model?*’ Accordingly, we first identified the research areas related to the model development: academic resilience, assessment, and test anxiety. Then, we examined narrative and scoping reviews and focus group discussions to find evidence in these areas.

##### Narrative reviews

We conducted three concurrent narrative reviews to collect sufficient scientific research for the model synthesis [46]. The first review identified the theoretical foundations and factors influencing academic resilience. The second review delineated the theoretical foundations of assessment in HPE. The third review identified the theories behind test anxiety. The articles included in this review were compiled into a table of evidence synthesis [47] (Appendix I) to extract key information regarding the theoretical foundations of academic resilience, test anxiety, and assessment systems and match them with the four resilience constructs [34].

##### Scoping review

Four of the authors (MW, MSBY, AFA, and NZ) conducted a scoping review to identify factors affecting test anxiety [48] following Arksey and O'Malley [49] stages of scoping reviews. Six electronic databases were used: PubMed, CINAHL, PsychINFO, ERIC (through EBSCOHST), SCOPUS, and ProQuest. The Preferred Reporting of Items for Systematic Reviews and Meta- Analyses (PRISMA) Statement [50] was followed to report the scoping review steps. Based on the factors identified in each study, codes from all studies were compiled to generate subthemes and overarching themes [48]. Appendix II contains a detailed description of the scoping review.

##### Focus group discussion (FGD)

Four of the authors (MW, MSBY, AFA, and NZ) conducted an FGD to elicit students’ knowledge, perspectives, and attitudes regarding test anxiety (TA) and their coping guidelines [51]. Appendix III contains a detailed description of the FGD steps and procedures.

### Phase II

#### Relating the identified constructs and their factors with each other

Phase 2 answered Whetten’s second question: ‘*How are these constructs/factors related to each other?*, and based on the notion of triangulation [45], the authors performed three subsequent steps:

- I. Based on the output of the scoping review and FGD, the factors decreasing TA were qualitatively analysed to generate guidelines for decreasing TA. Initially, similar factors were grouped together. Then, a suitable guideline was constructed capturing these factors. The initial guideline statements were revised to remove redundant statements and merge similar guidelines into a single statement. This iterative process was done until consensus was reached among the authors.
- II. Guided by the assessment cycle [52] and the sociotechnical model of assessment [53], the guidelines were thematically categorised into five phases of assessment. Each phase was named and defined.
- III. To evaluate the content validity of these guidelines, the authors utilised a structured tool called the Content Validity Index (CVI) [54, 55], which measures the proportional agreement when two or more expert panels independently evaluate the relevance of a model’s contents to the domain of interest. Ten experts, who were medical education and student assessment specialists, were invited to join the panels [54]. A four-point Likert scale was created in a Google form and sent to the experts via email. They were asked to evaluate the relevance of each guideline to its corresponding category (phase of assessment) [56]. An answer of 1 indicated the guideline was irrelevant, whereas an answer of 4 indicated that the item was extremely relevant.
- IV. Three indices were calculated: item/guideline-level CVIs (I-CVIs), scale-level CVIs (S-CVI) over the universal agreement method (S-CVI/UA), and the average calculation method (S-CVI/Ave) [54, 55, 57]. In I-CVIs, the relevance of each guideline to its related phase of assessment was evaluated by experts. Using a dichotomous rating of relevance, experts’ ratings of 1 or 2, indicating non-relevance, were counted as 0, while ratings of 3 and 4, indicating relevancy, were counted as 1 [57]. In S-CVI/UA, the proportion of guidelines receiving a rating of 3 or 4 (relevant) from all expert panels was calculated. In S-CVI/Ave, the mean I-CVI score for all guidelines was summed [57]. Appendix V contains the full content validity protocol.
- V. Based on these indices and the experts’ recommendations, five guidelines were removed, so the final set included 19 guidelines.

### Phase III

#### Sorting and ranking guidelines by experts

This phase answered Whetten’s third question: ‘Why is the proposed relationship represented by this portray?’ The same experts who were invited for content validation were asked to sort and rank each guideline into the appropriate four resilience constructs [58]. Using the checkboxes grid on the Google form, all of the guidelines were listed in one column, and the resilience constructs were placed at the top of the four adjacent columns. A column headed ‘not applicable’ was added for any guidelines that were irrelevant to the resilience constructs.

The responses were analysed using Microsoft Excel’s sorting and ranking functions. For each resilience construct, an Excel-based graphical representation was created based on the consensus of 50% of the experts if they categorised this particular guideline under a specific resilience construct.

Finally, using the Draw.io website, a conceptual map was created to link the guidelines-based assessment phases and resilience constructs to the theoretical foundation of phase I.

### Phase IV

#### Implication for practice

In phase IV, the authors aimed to answer Whetten’s final question: ‘*What are the implications of this model for research and practice?’* We evaluated the guidelines from the users’ perspective based on the response process method [59]. Twenty [20] participants were invited via email [60]. Apart from the invitation, the email contained a description of the research objectives and the validation process. A link to a video describing the application of SAR in assessment practice was also included. A response process form was attached to the email. The medical teachers were asked to review all guidelines and rate each of them based on its clarity and comprehensibility using a four-point scale (1 = not clear and comprehensible, 2 = somewhat clear and comprehensible, 3 = clear and comprehensible, 4 = very clear and comprehensible). Additionally, the participants were asked to provide written feedback on open-ended questions about the feasibility and applicability of the guidelines. Appendix VI contains the full content validity protocol.

We calculated three FVI indices: item/guideline FVIs (I-FVIs), scale/model FVIs using the universal agreement method (S-FVI/UA), and scale/model FVIs using the average method (S-FVI/UA) (Ave). First, all ratings were converted to a dichotomous scale: not clear (ratings of 1 and 2) and clear (ratings of 3 and 4). Then, we calculated the percentage of medical teachers who gave each guideline a ‘clear’ rating (I-FVIs). The proportion of guidelines receiving a rating of 3 or 4 (clear) from all medical teachers was then determined in S-FVI/UA. The average score of all I-FVIs for all guidelines was calculated in S-FVI/Ave. Based on these indices and the recommendations of the medical educators, two guidelines were eliminated, resulting in a final set of 17 guidelines.

### The final configuration of the framework

After all these phases, Microsoft Visio® was used to reshape the framework for the systematic assessment of resilience to make it more comprehensible and straightforward to implement. The four resilience constructs were placed in relation to the assessment phases, and the final list of phase-related guidelines was also placed.

## Results

### Phase I

The narrative reviews yielded relevant theoretical foundations in three areas: academic resilience, assessment, and test anxiety. These foundations were tabulated, and each relevant implication(s), through which resilience could be promoted, was identified (Table 1). In the first three columns, research areas, theoretical foundations/frameworks, and the implications of each study were presented, respectively. Guided by the integrated resilience model [34], every implication was matched to its suitable four resilience constructs: control, involvement, resourcefulness, and growth [34]. The matching was based on the agreement of the authors.

**Table 1:** Matching the implication of the identified theoretical frameworks with resilience constructs

			Resilience constructs [34]			
			Control	Involvement	Resourceful	Growth
Areas of narrative review	Theoretical frameworks	Implication(s) which can used to promote resilience				
Academic resilience	Motivation boosters and guzzlers model [33]	• Self-belief	**✓**			**✓**
		• Value of schooling			**✓**	
		• Learning persistence	**✓**			**✓**
		• Planning and monitoring	**✓**	**✓**	**✓**	**✓**
		• Study management				
		• Low anxiety	**✓**			
	The 5-C (confidence, coordination, control, composure and commitment) model of academic resilience. [61]	• Confidence, is synonymous with self-efficacy	**✓**			
		• Coordination, which means the ability of planning study tasks		**✓**		**✓**
		• Control, which refers to the ability to exert control over perceived uncertainty.	**✓**			
		• Composure, which indicates a low level of anxiety	**✓**			**✓**
		• Commitment, which entails keeping persistence.		**✓**		
	Coping reservoir tank [62]	• Psychosocial support			**✓**	
		• Social/healthy activities			**✓**	
		• Mentorship			**✓**	
		• Intellectual Stimulation	**✓**			**✓**
	Academic buoyancy process model [63]	• Increase self-efficacy	**✓**			
		• More engagement in the academic context		**✓**		
		• Low anxiety environment	**✓**			**✓**
		• More ability to control surroundings				**✓**
		• Good teacher-students relationship			**✓**	
	Higher-order model of resilience [64]	• Purpose in life	**✓**			**✓**
		• Self-esteem	**✓**			**✓**
		• Life satisfaction	**✓**			
		• Cognitive flexibility	**✓**			**✓**
		• Proactive coping		**✓**		**✓**
		• Social support			**✓**	
Assessment system	Utility of assessment [65]	• The educational effect should be considered as a means of improving students' learning and mental well-being.		**✓**		**✓**
	The supporting framework for student learning under assessment [66]	• Assessment expectations should be communicated clearly with students	**✓**	**✓**		
		• Sufficient feedback should be provided		**✓**		**✓**
		• Feedback should be focused on learning rather than on marks or students themselves		**✓**		**✓**
		• Feedback should be linked to the purpose of the task and to its criteria		**✓**		**✓**
		• Feedback should be understandable to students		**✓**		**✓**
	The wheel of competency assessment program [67]	• Assessment should have educational consequences (e.g. feedback)		**✓**		**✓**
		• Assessment should be meaningfulness (clear communication of what is expected and what done)	**✓**	**✓**		**✓**
		• A transparent communication of the assessment should be made.		**✓**		**✓**
	Good feedback practice model [68]	• The assessment process should assist in clarifying what constitutes good performance (goals, criteria, expected standards).		**✓**		
		• The assessment process should aid in the development of self-assessment (reflection) skills in the learning environment.		**✓**		**✓**
		• The assessment process should provide students with high-quality information about their learning progress.		**✓**		**✓**
		• The assessment process should promote dialogue between teachers and students about what they are learning.		**✓**	**✓**	**✓**
		• A positive assessment should promote positive motivational beliefs and self-esteem in the participants.	**✓**			**✓**
		• When a performance gap exists between current and desired performance, the assessment should identify ways to close it.		**✓**		**✓**
		• Teachers should be provided with information from the assessment that they can use to help shape their instruction.				
	A new model for designing programs of assessment [69]	• Assessment drives learning		**✓**		**✓**
	Criteria of good assessment [70]	• The educational effect should be considered as a means of improving students' learning and mental well-being.	**✓**	**✓**		**✓**
	A model of the pre-assessment learning effects on student assessment. [71]	• Improving learning outcomes through assessment design that takes into account impact appraisal, or the student's appraisal of the assessment	**✓**	**✓**		**✓**
		• Designing assessments to promote learning while taking response appraisal into consideration		**✓**		**✓**
		• Assessment should be designed to promote learning and take into account perceived agency.		**✓**	**✓**	
		• Consideration of interpersonal factors in the design of assessments to promote learning		**✓**		**✓**
	Aspire criteria for assessment [72]	• Learning opportunities should be supported, enhanced, and created through the assessment programme.		**✓**		**✓**
Test anxiety	The cognitive attentional model [73, 74]	• Students’ cognitive reactions can be better controlled by improving their own perception, which will reduce test anxiety.	**✓**			**✓**
		• Students' emotional reactions can be controlled by managing their interactions on exam day.	**✓**			**✓**
	The learning deficit model [75]	• Assistance in improving preparation will help students to feel less anxious about tests.		**✓**	**✓**	
	The dual deficit model [76]	• All the previous implications are applied here as this theory was built on them.	**✓**	**✓**	**✓**	**✓**
	Self-regulation model [77]	• Help student to set goals through good feedback and continuous monitoring	**✓**			**✓**
	Self-Worth Model [78]	• Improving the positive self-image of students by dealing with them in a temperate manner in all situations, particularly during examinations.		**✓**	**✓**	
	Transactional Process Model [79]	• Interventions to improve the impact of each of the following will reduce test anxiety: Students psychological effects	**✓**			**✓**
		• Interventions to improve the impact of each of the following will reduce test anxiety: Environmental situations		**✓**	**✓**	

The scoping review revealed that factors related to test anxiety can be categorised into two groups: those that increase TA and those that decrease TA (Appendix II). Likewise, the thematic analysis of the medical student focus group discussion yielded three major themes: students, academic resources, and examiners. Each theme was subdivided into subthemes that corresponded to an increase or decrease in TA [51] (Appendix III).

### Phase II

#### Compiling and categorizing the generated guidelines

Based on the scoping review and FGD findings, the authors compiled a list of test anxiety-reducing guidelines (Appendix IV). The list was evaluated, and guidelines that were duplicated were eliminated. Guided by the assessment cycle [52] and the model of ‘assessment as a sociotechnical system’ [53], the authors categorised the generated guidelines into groups and subsequently named and defined each group. The following five phases of evaluation were identified:

- 1. Assessment direction, which focuses on improving the candidate’s understanding of the assessment’s scope and procedure.
- 2. Assessment preparation, which emphasises enhancing the candidate’s cognitive, mental, and psychomotor readiness to optimise assessment performance.
- 3. Assessment experience, which enhances the formative assessment component.
- 4. Examiner focus, which relates to improving examiner behaviour to improve the candidate’s performance and decrease the candidate’s anxiety.
- 5. Student reflection, which encourages reflection by students.

Table 2 shows each phase and its related guidelines.

**Table 2:** Categorization of SAR guidelines based on stages and phases of assessment

Stage of assessment	Classified guidelines to enhance resilience
Pre-Assessment “Anticipation and Preparation:	**Assessment direction: Improving candidate's knowledge of the assessment scope and process** 1. Share of assessment mapping whenever applicable 2. Sharing of assessment rubric in modalities whenever applicable 3. Briefing on the overall assessment coverage 4. Establish a briefing session before exam 5. Familiarize students with assessment methods **Assessment preparation: Improving candidates cognitive, mental, and psychomotor preparedness to maximize assessment performance** 6. Advice students about study skills 7. Advice students about time management [80–83] 8. Direct students for good material for revision 9. Advice students on exam skills 10. Brief students about the grading system 11. Provide guidelines for students to reduce test anxiety 12. Provide self-care guidelines to students before assessment **Increase assessment experience: Enriching the formative component of the assessment** 13. Increase frequency of formative assessment 14. Increase continuous assessment 15. Encourage to have targeted mock exams 16. Promote/encourage collaborative assessment 17. Promote open book exam 18. Use peer assessment 19. Introduce progress testing
Intra-assessment	**Examiner focus: Improving examiner behavior to maximize candidate performance reduce candidate anxiety** 20. Establish non-threatening environment during exam: smiling face, welcoming, professional behavior, rapport, sense of humor [84–88]
Post-Assessment Reflection and feedback	**Student reflection: promote student’s reflection** 21. Add free space in the written assessment to get students feedback 22. Add free space in the written assessment to write self-reflection 23. Increase feedback by examiner 24. Sharing the key answer if applicable

#### Content validation of the guidelines

Six of the 10 expert panels responded to the invitation to participate in the content validation process. The expert panels had extensive experience in medical education, student assessment, and psychological well-being (Appendix V). Following the principles for calculating CVIs [55, 57], all items (guidelines) with an I-CVI of 0.83 or higher were deemed relevant and included in the response process study based on the CVI. A global acceptance level of 0.80 or higher for S-CVI/UA and S-CVI(Ave) indicates that all components are relevant to the framework. Before the subsequent step, modifications were made based on feedback, which resulted in the elimination of items scoring less than 0.83. Table 3 shows the list of guidelines based on the content validation.

**Table 3:** The CVIs of the guidelines after removing four items

SAR guidelines (*n* = 19)	Expert 1	Expert 2	Expert 3	Expert 4	Expert 5	Expert 6	Experts in agreement		I-CVI	UA
1. Sharing of assessment mapping whenever applicable	1	1	1	1	1	1	6		1.0	1
2. Sharing of assessment rubric in modalities whenever applicable	1	1	1	1	1	1	6		1.0	1
3. Briefing on the overall assessment coverage	1	1	1	0	1	1	5		0.8	1
4. Establishing a briefing session before exam	1	1	1	1	0	1	5		0.8	1
5. Familiarizing students with assessment methods	1	1	1	1	1	1	6		1.0	1
6. Increasing frequency of formative assessment	1	1	1	1	1	1	6		1.0	1
7. Encourage to have targeted mock exams	1	1	1	1	1	0	5		0.8	1
8. Promoting/encouraging collaborative assessment	1	1	1	1	1	1	6		1.0	1
9. Promoting open book exam	1	1	1	1	1	1	6		1.0	1
10. Using peer assessment	1	1	1	1	0	1	5		0.8	1
11. Introducing progress testing	1	1	1	1	0	1	5		0.8	1
12. Advising students about time management	1	1	1	1	0	1	5		0.8	1
13. Directing students for good material for revision	1	1	1	1	1	1	6		1.0	1
14. Advising students on exam skills	1	1	1	1	0	1	5		0.8	1
15. Providing guidelines for students to reduce test anxiety	1	1	1	1	1	1	6		1.0	1
16. Establishing non-threatening environment during exam: smiling face, welcoming, professional behavior, rapport, sense of humor	1	1	1	0	1	1	5		0.8	1
17. Adding free space in the written assessment to write self-reflection	1	1	1	1	0	1	5		0.8	1
18. Increasing feedback by examiner	1	1	1	1	0	1	5		0.8	1
19. Sharing the key answer if applicable	1	1	1	1	1	1	6		1.0	1
Total of items	19	19	19	17	12	18		**S-CVI/Ave**	**0.91**	
Proportional relevance	1	1	1	0.89	0.63	0.95		**S-CVI/UA**		**1.0**
**S-CVI/Ave**	**0.91**									

**Fig. 2:**
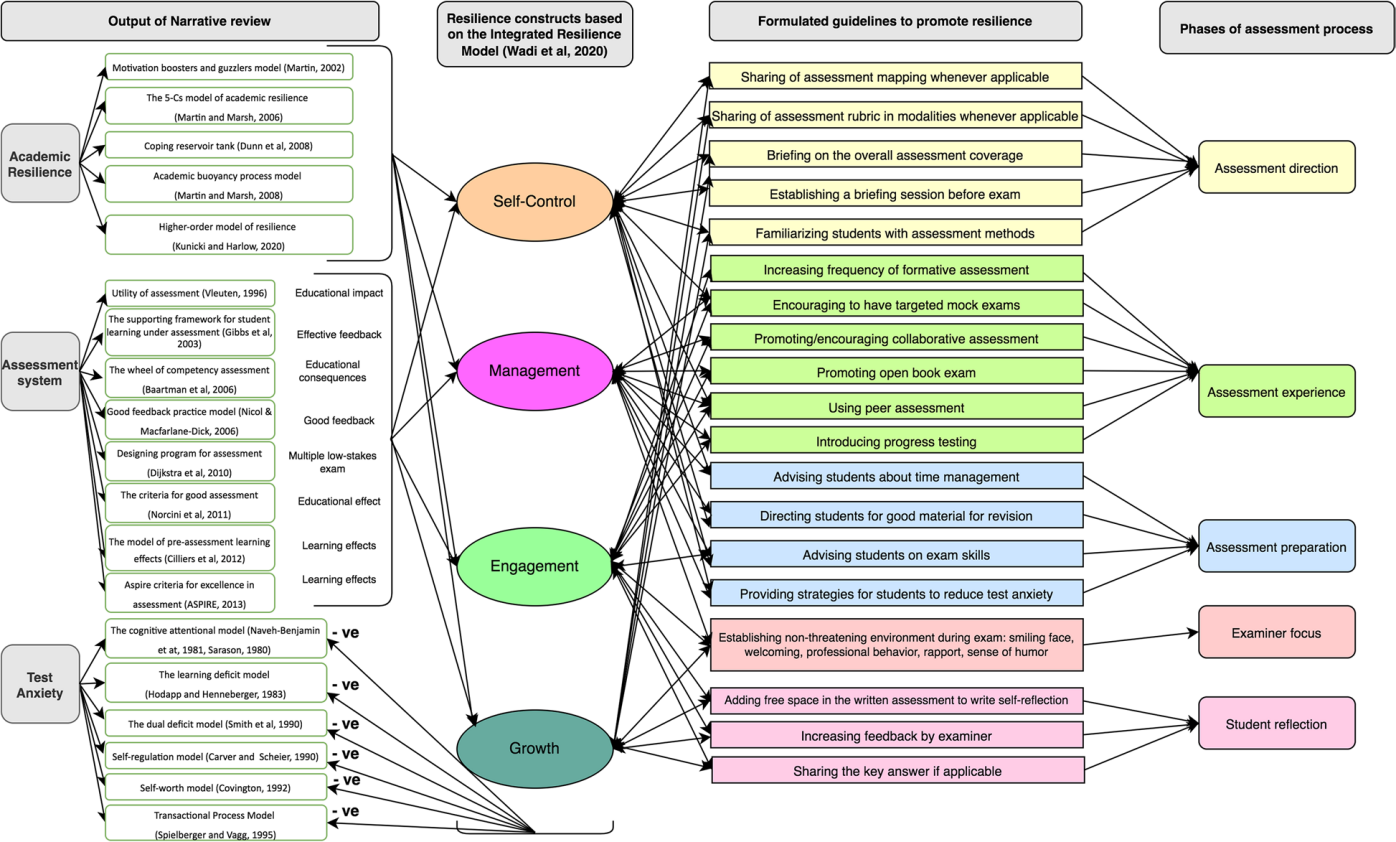
Mapping phase I output with the SAR framework and its guidelines

Figure 2 illustrates the correlation and configuration of the relationship between the findings of phase I of the study. The right side of Fig. 2 presents the recommended guidelines for fostering resilience through five phases of assessment. They are connected to the four resilience constructs with arrows that point in both directions to illustrate the reciprocal relationship between guidelines and resilience constructs. The four resilience constructs were linked to the theoretical foundations of academic resilience and the assessment system, and they were shown to have an inverse relationship with test anxiety.

### Phase III

**Fig. 3:**
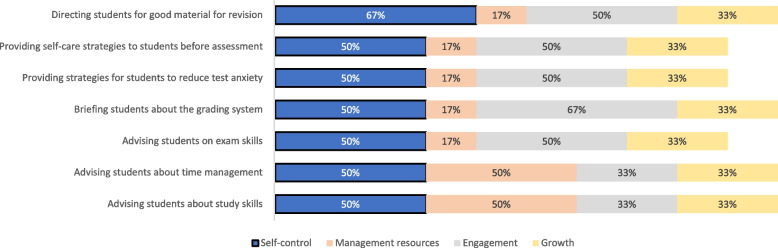
Sorting and ranking of guidelines relating “self-control” construct

**Fig. 4:**
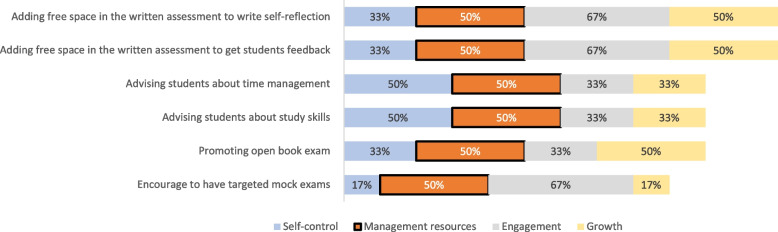
Sorting and ranking of guidelines relating “management of resources” construct

**Fig. 5:**
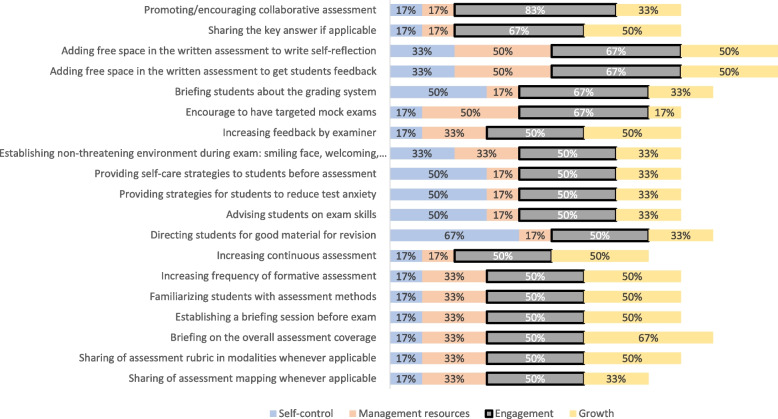
Sorting and ranking of guidelines relating “engagement” construct

**Fig. 6:**
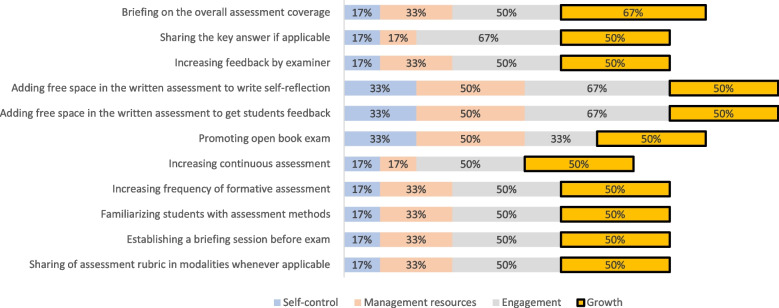
Sorting and ranking of guidelines relating “growth” construct

Figures 3, 4, 5, and 6 display the results of the experts’ ranking and sorting. Each figure represents a resilience construct and its corresponding guidelines, on which 50% of the experts agreed.

### Phase IV

The response process aimed to evaluate the use of SAR and its guidelines among medical teachers. Twelve [12] of the 20 invited panels responded to the invitation. The panels consisted of medical teachers from various disciplines and universities (Appendix VI). On the basis of the FVI [60], 17 items (guidelines) with an I-FVI of 0.83 or higher were retained, indicating their relevance, while two guidelines with scores below the threshold were eliminated (two guidelines). Table 4 presents the final list of guidelines. As a result, the overall framework S-FVI/UA and S-FVI (Ave) improved from 0.92 to 0.94, indicating the clarity and understandability of all framework components (Table 4).

**Table 4:** The FVI indices of SAR after removing some guidelines

SAR guidelines (*n* = 17)	**Expert 1**	**Expert 2**	**Expert 3**	**Expert 4**	**Expert 5**	**Expert 6**	**Expert 7**	**Expert 8**	**Expert 9**	**Expert 10**	**Expert 11**	**Expert 12**	Experts in agreement		**I-CVI**	UA
1. Sharing of assessment mapping/blueprinting whenever applicable	1	1	1	1	1	1	1	1	1	1	1	1	**12**		**1.00**	**1**
2. Sharing of assessment rubric in modalities whenever applicable	1	1	1	1	1	0	1	1	1	1	1	1	**11**		**0.92**	**1**
3. Briefing on the overall assessment coverage	1	1	1	1	1	1	1	1	1	1	1	1	**12**		**1.00**	**1**
4. Establishing a briefing session before exam	1	1	1	1	1	1	1	1	1	1	1	1	**12**		**1.00**	**1**
5. Familiarizing students with assessment methods	1	1	1	1	1	1	1	1	1	1	1	1	**12**		**1.00**	**1**
6. Advising students about time management and study skills	1	1	1	1	1	1	1	1	1	1	1	1	**12**		**1.00**	**1**
7. Directing students for good material for revision	1	1	1	1	1	0	1	1	1	1	1	1	**11**		**0.92**	**1**
8. Advising students on exam skills	1	1	1	1	1	1	1	1	1	1	1	1	**12**		**1.00**	**1**
9. Providing guidelines for students to reduce test anxiety	0	1	1	1	0	1	1	1	1	1	1	1	**10**		**0.83**	**1**
10. Increasing frequency of formative assessment	1	1	1	1	1	1	1	1	1	1	1	1	**12**		**1.00**	**1**
11. Encourage to have targeted mock exams	1	1	1	1	1	1	1	1	1	1	1	1	**12**		**1.00**	**1**
12. Promoting/encouraging collaborative assessment	1	1	1	1	0	1	0	1	1	1	1	1	**10**		**0.83**	**1**
13. Promoting open book exam	1	1	1	1	0	1	0	1	1	1	1	1	**10**		**0.83**	**1**
14. Using peer assessment	1	1	1	1	0	0	1	1	1	1	1	1	**10**		**0.83**	**1**
15. Establishing non-threatening environment during exam	1	1	1	1	1	1	1	1	1	1	1	1	**12**		**1.00**	**1**
16. Increasing feedback to examinee	1	1	1	1	1	1	1	1	1	1	1	1	**12**		**1.00**	**1**
17. Adding free space/window for self-reflection	1	1	1	1	0	1	0	1	1	1	1	1	**10**		**0.83**	**1**
Total of items	**16**	**17**	**17**	**17**	**12**	**14**	**14**	**17**	**17**	**17**	**17**	**17**		**S-CVI/Ave**	**0.94**	
Proportional relevance	**0.94**	**1.00**	**1.00**	**1.00**	**0.71**	**0.82**	**0.82**	**1.00**	**1.00**	**1.00**	**1.00**	**1.00**		**S-CVI/UA**		**1.0**
**S-CVI/Ave**	**0.94**															

**Fig. 7:**
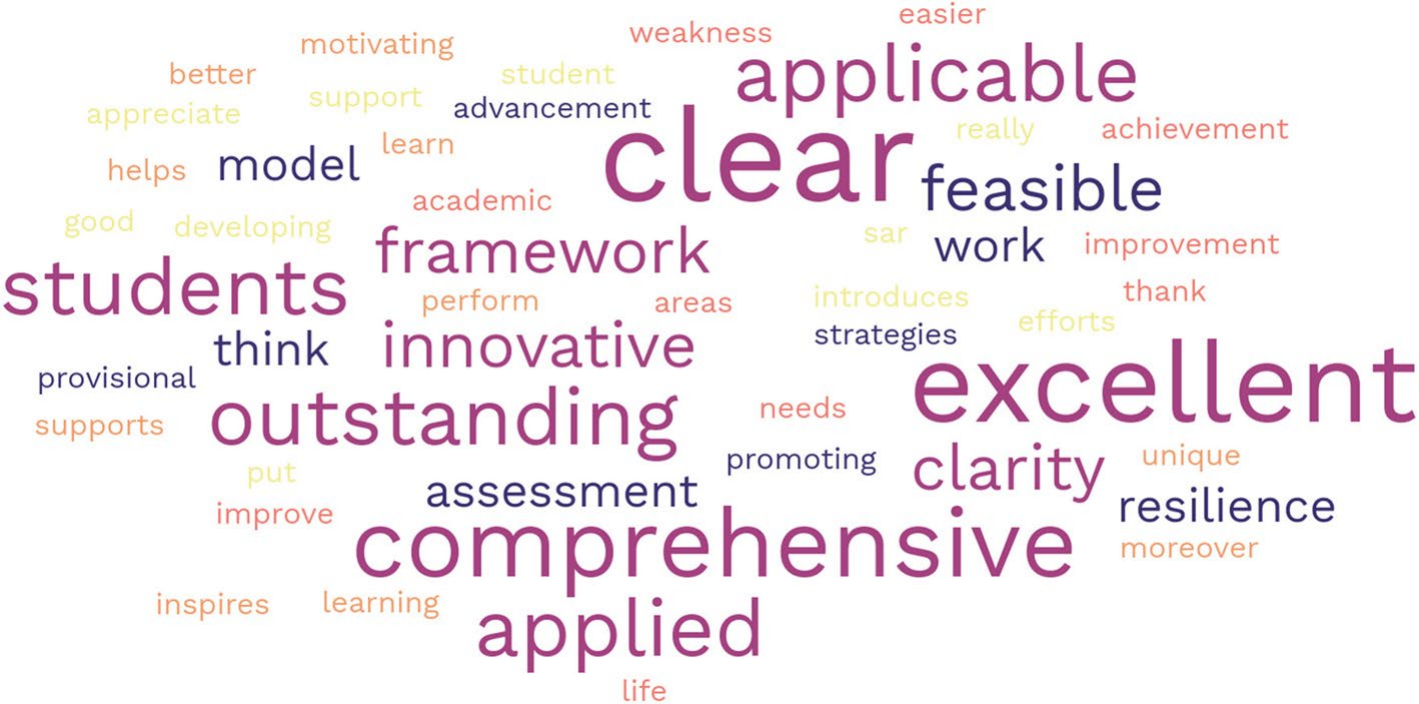
Word cloud of the most common comments made by medical educators

The medical teachers also gave encouraging written insights about the guidelines and their clarity (Appendix VII), which are summarised as a word cloud in Fig. 7.

### The SAR framework

**Fig. 8:**
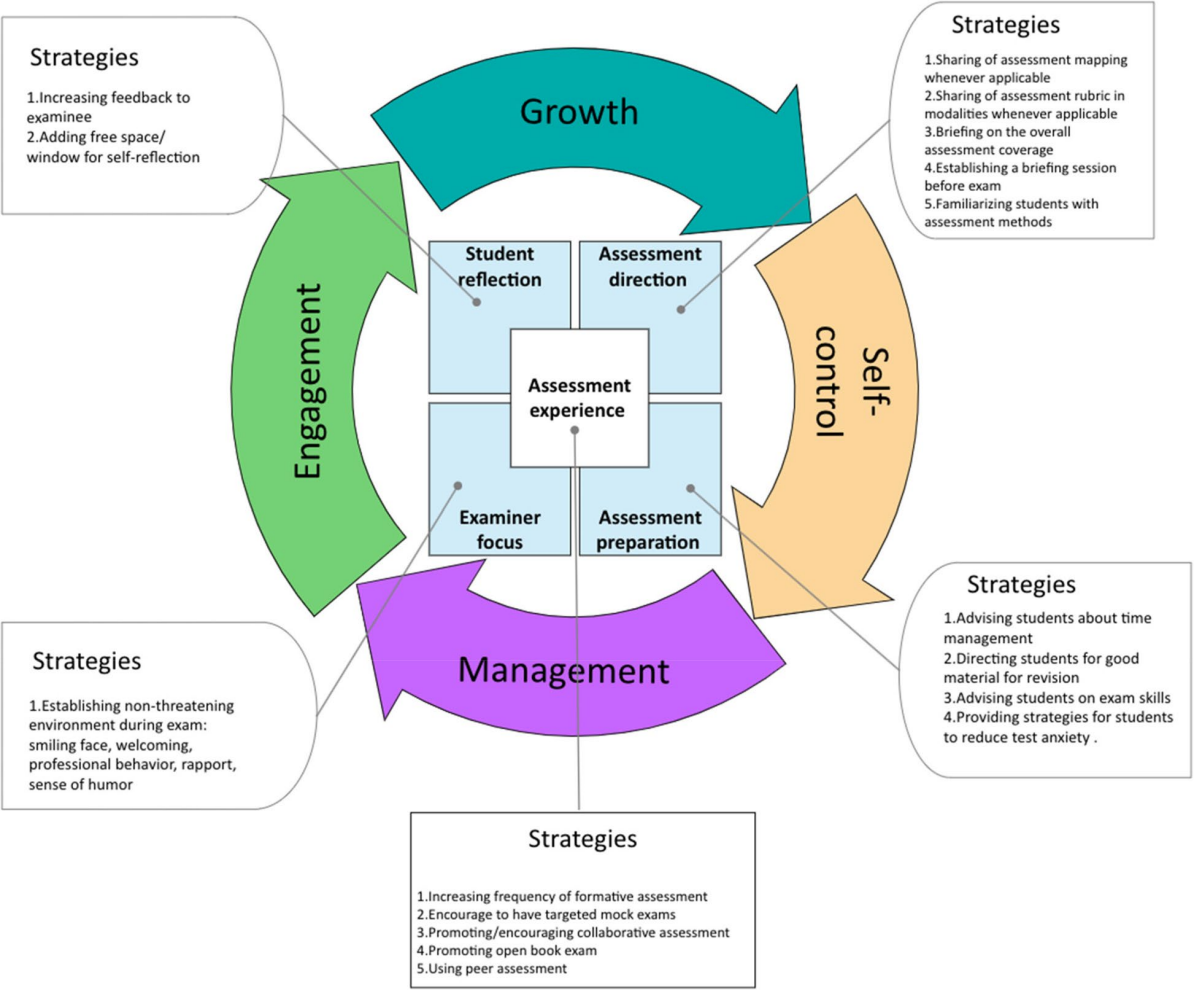
The Systematic Assessment for Resilience (SAR) framework

The SAR framework (Fig. 8) incorporates the overarching relationship between the four resilience constructs [34], the five phases of the assessment process, and their relevant strategies for promoting resilience. The four constructs of resilience include 1) self-control, meaning that students should be able to govern themselves and face adversity, 2) management, which describes students’ ability to use available resources effectively to overcome obstacles, 3) engagement, which refers to students’ ability to be involved and committed to pursuing the challenge, and 4) growth, which reflects students’ ongoing development to face future challenges. The four constructs are part of an ongoing continuous cycle.

The ‘assessment experience’ phase is represented as the nucleus of the framework, as it is the core of the assessment process. In this phase, students’ resilience may be promoted through various strategies, such as increasing the frequency of formative assessment, encouraging targeted mock exams  [87–91], promoting collaborative assessment and open book exams, peer assessment, and introducing progress testing. In the ‘assessment direction’ phase, students’ knowledge of the assessment scope and process may be improved through various strategies, including the sharing of assessment mapping and the assessment rubric, briefing on the overall assessment coverage, establishing a briefing session before the exam, and familiarising students with the assessment methods. Such strategies will foster resilience, especially the self-control and growth constructs. In the ‘assessment preparation’ phase, students’ cognitive, mental, and psychomotor preparedness are improved to maximise their assessment performance. Various strategies may be used, such as advising students on study skills, time management, and exam skills, directing students to good materials for revision, and providing strategies for students to reduce test anxiety. These strategies will promote resilience, especially the self-control and management constructs. The ‘examiner focus’ assessment phase deals with examiners’ behaviour to maximise students’ performance and reduce their anxiety. This phase can be improved by establishing a non-threatening environment during examinations, for example, by smiling, engaging in welcoming and professional behaviour, building rapport, and showing a sense of humour. All of these will foster resilience, especially the engagement and management constructs. The ‘student reflection’ assessment phase promotes students’ reflection by providing space in the written assessment for students to offer feedback on the examiner. Such strategies will improve students’ resilience, particularly in the engagement and growth constructs.

## Discussion

As the prevalence of pathological stress among medical and HPE students continues to rise [3–8], a number of interventional programmes have been designed to improve their mental health [15]. The primary criticism of these programmes is their emphasis on causes other than the primary cause, which is the assessment. This study provides a methodical approach for promoting resilience while practicing the assessment. A variety of strategies promote resilience through the five phases of assessment. Resilience is comprised of four constructs, which are shown in Fig. 8. In the following sections, we will discuss each assessment phase and how it can promote resilience.

### Assessment experience

The assessment experience phase is purposively located at the centre of this framework, as it promotes the four resilience constructs. The assessment experience emphasises the frequency of formative assessment or any other assessment experience (e.g. mock exam), offering students an opportunity to engage in a simulated challenge similar to the real assessment. Such an experience creates a space for self-regulation and thus enhances self-efficacy and learning growth [92]. There is a growing body of research highlighting the crucial role of formative assessment in maximising learning [93, 94]. Formative assessment serves as a tool for practicing assessment, reflecting on performance, and identifying weak points and opportunities to improve actual future performance [68]. In this study, the strategies within the assessment experience were designed to maximise self-regulatory learning and evaluative judgment [92, 93]. In a nutshell, the more exposure to the assessment experience, the greater the options for strengthening evaluative judgment and hence self-regulation, which is expressed as ‘*self-control*’ in our framework. Furthermore, increasing ‘assessment experience’ will lead students to the focus on the prudent use of available resources, ‘*management’*, vividly experiencing the actual assessment, ‘*engagement’*, and self-esteem—in other words, on ‘*growth*’ (Fig. 8).

### Assessment direction

The current assessment practice in the medical and HPE fields is competency-based assessment [95–97]. Consequently, it is crucial to communicate with students clearly about these competencies and how they will be evaluated [98]. The ‘assessment direction’ involves directing students towards assessment by providing them with clear instructions on how the assessment will be administered and what is expected of them. Knowing what is expected of them during assessment will enable them to shape their learning and direct their efforts to achieve these objectives [99]. A considerable body of studies has shown that defining the assessment expectations (objectives) will allow students to target their efforts to achieve them [98, 100]. However, there is an issue in terms of which assessment criteria and standards should be communicated to students. There is no research consensus on the suitable methods for communicating with students. In our framework, we provide a variety of strategies examiners could use to direct students toward assessment.

Articulating the assessment direction has benefits for both the examinee and examiner. The examinee can then tailor his or her efforts to the assessment direction. The examiner can select suitable assessment modalities to measure the desired outcomes [96]. Additionally, it reduces the burden on examiners related to answering examinees’ questions about exams. Moreover, examinees’ test anxiety will be reduced, and they will have more agency in meeting the expectations [101]. In summary, directing students toward assessment will enhance their self-control, supporting self-regulated learning [93], and empower them to grow and face future challenges [100].

### Assessment preparation

When guiding students in the assessment direction, it is important to ensure that they have the proper tools to succeed. Hence, assessment preparation plays an important role in meeting the challenge of assessment. Studies have shown that helping students control their negative thoughts and advising them on learning skills and time management will maximise their learning [102, 103]. In our framework, we believe that teachers play the central role in maximising learners’ behaviour. In addition to preparing high-quality learning materials, teachers can also provide students with rich resources to improve their mental well-being and reduce the negative effects of assessment. This practice will enhance students’ self-control and shift their mindset so they can use the resources around them to face assessment challenges.

### Examiner focus

This framework highlights the examiner’s conduct during the exam. The direct encounter between examiner and examinee has a psychological dimension, creating life-altering memories that can either destroy or reinforce the examinee’s self-esteem and, consequently, resilience. While the presence of an examiner in the examination room automatically causes test anxiety, the situation will become worse if there is a lack of proper communication or if the examiner chooses to fail students based on personal preferences or biases [51]. This negative situation has been dubbed ‘the hawk effect’ [104]. Our framework proposes techniques to mitigate this unintended consequence and foster an atmosphere conducive to reciprocal communication and learning, which automatically enhances students’ ‘engagement’ and prepares them for similar situations in the future by managing learning resources wisely and effectively.

### Student reflection

Self-assessment (or self-reflection) has been proven to be an effective approach to support learning engagement. In self-reflection, students evaluate their performance related to both internally set goals and externally set criteria [105]. In this framework, teachers (assessors) provide systematised avenues for self-reflection to achieve the desired resilience construct: growth. While several studies have presented different approaches for promoting self-reflection, we encourage assessors to use the most common reflection method: feedback [100]. Nicol and Macfarlane‐Dick [68] described the most important aspect of high-quality feedback: ‘Good quality external feedback is information that helps students troubleshoot their own performance and self-correct; that is, it helps students take action to reduce the discrepancy between their intentions and the resulting effects’ [68]. Numerous studies on HPE have promoted the use of feedback [106], and it is key component in the programmatic assessment framework [107]. Both feedback and self-reflection support each other to maximise learning and hence ‘growth’ [108]. Through this feedback, students will receive constructive comments regarding their performance based on the teacher’s expectation or established criteria, which they can then use internally to redesign a suitable learning path to achieve their goals [100]. Studies have shown that students’ self-reflection leads to deep learning, self-efficacy, self-regulation, and personal growth [93, 106]. While some researchers argue that students should be trained in self-regulation, others contend that self-regulation is a spontaneous process that can be maximised by providing a suitable platform to practice it [109]. Consequently, the framework provides additional resources for assessors to promote self-reflection.

### Limitations and future perspectives

During the literature review phase of developing the SAR framework, efforts were made to broaden the search of the scoping review to include literature in HPE rather than just medicine, and to conduct narrative reviews that considered higher education in general. However, the FGD only included one medical school. Another limitation of this research was that it only included articles written in English, which may introduce bias (also known as language bias [110]) and result in the omission of important cultural contexts and necessary details in data synthesis. However, the triangulation of the findings with those from the scoping review, other narrative reviews, and the FGD mitigated the aforementioned limitations [45].

## Conclusions

Resilience is proven to be the desired construct for medical and health professions students. It fosters several characteristics graduates need to meet future challenges and adversities. The current study presents a systematic method for fostering student resilience through assessment practice. Based on rigorous methodological research and a theoretical foundation, the study provides a set of practical strategies for promoting resilience. Through five phases of assessment, namely, assessment direction, assessment preparation, assessment experience, examiner focus, and student reflection, the SAR framework aims to promote four resilience constructs: self-control, management, engagement, and growth.

## Supplementary Information

**Additional file 1: Appendix I.** (Three narrative reviews). **Table S1.** The identified theoretical frameworks of academic resilience. **Table S2.** The identified theoretical frameworks of assessment. **Table S3.** The identified theoretical frameworks of test anxiety. **Appendix II.** (Scoping review). **Figure S1.** PRISMA chart of the scoping review identifying factors related to test anxiety. **Table S3.** Descriptive variables of the included studies (*n* = 74). **Table S4.** Descriptive variables of the included studies (*n* = 74). **Table S5.** Themes of factors that increase test anxiety. **Table S6.** Themes of factors that decrease test anxiety. Appendix III. (Focus Group Discussion). **Figure S2.** Emerged themes and sub-themes in relation to increasing and decreasing test anxiety. **Table S7.** Emerged themes and sub-themes from FGD with supporting quotations. **Appendix IV.** (Generating resilience guidelines based on the scoping review and FGD ). **Figure S3.** Relation of the proposed guidelines with outputs of scoping review and FGD. **Appendix V.** (Content Validation). **Table S8.** Content Validation Index - Guidelines rated as 3 or 4 (relevant) is ticked on the table. **Table S9.** Acceptable values for content validity indices (104). **Table S10.** Demographic data of the expert panels. **Table S11.** The original version of the SAR guidelines that was sent for content validation. **Appendix VI.** (Response Process). **Table S12.** Content Validation Index - Guidelines rated as 3 or 4 (relevant) is ticked on the Table S. **Table S13.** Demographic data of the panels in face validation study. **Table S14.** The FVI indices of the 19 SAR guidelines. **Appendix VII**. (Written response of medical teacher in using SAR guideline). **Table S15.** The medical teachers’ feedback after response process.

## Data Availability

Please email the corresponding author for a link to the de-identified datasets. However, due to privacy concerns, the FGD transcripts are unavailable to the general public.

## Abbreviations

CVI: Content Validity Index
FGD: Focus Group Discussion
FVI: Face Validity Index
HPE: Health Professions Education
SAR: Systematic Assessment for Resilience
TA: Test Anxiety

## References

[1] Veal CT (2021). We burn out, We break, We die: medical schools must change their culture to preserve medical student mental health. Acad Med.

[2] Tian-CiQuek T, Tam W-S, Tran BX, Zhang M, Zhang Z, Su-HuiHo C (2019). The global prevalence of anxiety among medical students: a meta-analysis. Int J Environ Res Public Health.

[3] Erschens R, Keifenheim KE, Herrmann-Werner A, Loda T, Schwille-Kiuntke J, Bugaj TJ (2019). Professional burnout among medical students: systematic literature review and meta-analysis. Med Teach.

[4] Frajerman A, Morvan Y, Krebs M-O, Gorwood P, Chaumette B (2019). Burnout in medical students before residency: a systematic review and meta-analysis. Eur Psychiatr.

[5] Abdalla ME, Shorbagi S (2018). Challenges faced by medical students during their first clerkship training: a cross-sectional study from a medical school in the Middle East. J Taibah Univ Med Sci.

[6] Jordan RK, Shah SS, Desai H, Tripi J, Mitchell A, Worth RG (2020). Variation of stress levels, burnout, and resilience throughout the academic year in first-year medical students. PLoS ONE.

[7] Ragab EA, Dafallah MA, Salih MH, Osman WN, Osman M, Miskeen E (2021). Stress and its correlates among medical students in six medical colleges: an attempt to understand the current situation. Middle East Curr Psychiatr.

[8] Moir F, Yielder J, Sanson J, Chen Y (2018). Depression in medical students: current insights. Adv Med Educ Pract.

[9] Ribeiro ÍJS, Pereira R, Freire IV, de Oliveira BG, Casotti CA, Boery EN (2018). Stress and quality of life among university students: a systematic literature review. Health Prof Educ.

[10] Flaherty JA, Richman JA (1993). Substance use and addiction among medical students, residents, and physicians. Psychiatr Clin North Am.

[11] Hays LR, Cheever T, Patel P (1996). Medical student suicide, 1989–1994. Am J Psychiatry.

[12] Newbury-Birch D, White M, Kamali F (2000). Factors influencing alcohol and illicit drug use amongst medical students. Drug Alc Depend.

[13] Pickard M, Bates L, Dorian M, Greig H, Saint D (2000). Alcohol and drug use in second-year medical students at the university of leeds. Med Educ.

[14] Dyrbye LN, Thomas MR, Shanafelt TD (2006). Systematic review of depression, anxiety, and other indicators of psychological distress among U.S. and Canadian medical students. Acad Med.

[15] Yusoff MSB (2014). Interventions on medical students’ psychological health: a meta-analysis. J Taibah Univ Med Sci.

[16] Yusoff MSB, Rahim AFA, Yaacob MJ (2010). Prevalence and sources of stress among universiti sains Malaysia medical students. Malaysian J Med Sci: MJMS.

[17] Yusoff MSB, Yee LY, Wei LH, Siong TC, Meng LH, Bin LX, et al. A study on stress, stressors and coping strategies among Malaysian medical students. International Journal of Students' Research. 2011;1(2) (Retrieved from: http://www.ijsronline.net/article.asp?issn=2321-6662;year=2011;volume=1;issue=2;spage=45;epage=50;aulast=Yusoff;type=0)).

[18] Yusoff MSB. Impact of summative assessment on first year medical students’ mental health. International Medical Journal. 2011;18(3):172–5. (Retrieved from: https://www.researchgate.net/publication/215632306_Impact_of_Summative_Assessment_on_First_Year_Medical_Students'_Mental_Health).

[19] Aziz N, Serafi AH. Increasing Levels of Test Anxiety and Psychological Distress with Advancing Years of Medical Education. British Journal of Medical and Health Research. 2017;4(3):(Retrieved from: https://www.researchgate.net/publication/315830384_Increasing_Levels_of_Test_Anxiety_and_Psychological_Distress_with_Advancing_Years_of_Medical_Education#fullTextFileContent).

[20] Boparai JK, Gupta AK, Singh A, Matreja PS, Khanna PML, Garg P. Impact of test anxiety on psychomotor functions and satisfaction with life of medical undergraduates during second professional curriculum. Education in Medicine Journal. 2013;5(4):e6-e11. 10.5959/eimj.v5i4.172

[21] Wald HS (2020). Optimizing resilience and wellbeing for healthcare professions trainees and healthcare professionals during public health crises–Practical tips for an ‘integrative resilience’approach. Med Teach.

[22] Bonanno GA (2004). Loss, trauma, and human resilience: have we underestimated the human capacity to thrive after extremely aversive events?. Am Psychol.

[23] Bonanno GAP, Mancini ADP (2008). The human capacity to thrive in the face of potential trauma. Pediatrics.

[24] Nucifora F, Langlieb AM, Siegal E, Everly GS, Kaminsky M (2007). Building resistance, resilience, and recovery in the wake of school and workplace violence. Disast Med Public Health Prep.

[25] Zwack J, Schweitzer J (2013). If every fifth physician is affected by burnout, what about the other four? Resilience strategies of experienced physicians. Acad Med.

[26] Schwarz S (2018). Resilience in psychology: a critical analysis of the concept. Theory Psychol.

[27] Sergeant J, Laws-Chapman C (2012). Creating a positive workplace culture. Nurs Manage (through 2013).

[28] Alva SA (1991). Academic invulnerability among Mexican-American students: the importance of protective resources and appraisals. Hispanic J Behav Sci.

[29] Ye W, Strietholt R, Blömeke S (2021). Academic resilience: underlying norms and validity of definitions. Educ Assess, Eval Accountabil.

[30] García-Crespo FJ, Fernández-Alonso R, Muñiz J (2021). Academic resilience in European countries: the role of teachers, families, and student profiles. PLoS ONE.

[31] Huey CWT, Palaganas JC (2020). What are the factors affecting resilience in health professionals? A synthesis of systematic reviews. Med Teach.

[32] Martin A (2002). Motivation and academic resilience: developing a model for student enhancement. Aust J Educ.

[33] Wadi M, Nordin NI, Syazni N, Roslan TC, Yusoff MSB (2020). Reframing resilience concept: insights from a meta-synthesis of 21 resilience scales. Educ Med J..

[34] Cooke GP, Doust JA, Steele MC (2013). A survey of resilience, burnout, and tolerance of uncertainty in Australian general practice registrars. BMC Med Educ.

[35] Fox S, Lydon S, Byrne D, Madden C, Connolly F, O’Connor P (2018). A systematic review of interventions to foster physician resilience. Postgrad Med J.

[36] Sood A, Sharma V, Schroeder DR, Gorman B (2014). Stress Management and Resiliency Training (SMART) program among department of radiology faculty: a pilot randomized clinical trial. EXPLORE: J Sci Healing.

[37] Mache S, Baresi L, Bernburg M, Vitzthum K, Groneberg D (2017). Being prepared to work in gynecology medicine: evaluation of an intervention to promote junior gynecologists professionalism, mental health and job satisfaction. Arch Gynecol Obstetr.

[38] Pliego JF, Wehbe-Janek H, Rajab MH, Browning JL, Fothergill RE (2008). OB/GYN boot camp using high-fidelity human simulators: enhancing residents’ perceived competency, confidence in taking a leadership role, and stress hardiness. Simul Healthc.

[39] Runyan C, Savageau JA, Potts S, Weinreb L (2016). Impact of a family medicine resident wellness curriculum: a feasibility study. Med Educ Online.

[40] Sood A, Prasad K, Schroeder D, Varkey P (2011). Stress management and resilience training among department of medicine faculty: a pilot randomized clinical trial. J Gen Internal Med.

[41] Joyce S, Shand F, Tighe J, Laurent SJ, Bryant RA, Harvey SB (2018). Road to resilience: a systematic review and meta-analysis of resilience training programmes and interventions. BMJ Open.

[42] van Kessel G, Brewer M, Lane M, Cooper B, Naumann F (2022). A principle-based approach to the design of a graduate resilience curriculum framework. Higher Educ Res Dev.

[43] Whetten DA (1989). What constitutes a theoretical contribution?. Acad Manage Rev.

[44] Hussein A (2009). The use of triangulation in social sciences research: can qualitative and quantitative methods be combined?. J Compar Soc Work..

[45] Finfgeld-Connett D (2010). Generalizability and transferability of meta-synthesis research findings. J Adv Nurs.

[46] Green BN, Johnson CD, Adams A (2006). Writing narrative literature reviews for peer-reviewed journals: secrets of the trade. J Chiropractic Med.

[47] Wadi M, Yusoff MSB, Abdul Rahim AF, Lah NAZN (2022). Factors influencing test anxiety in health professions education students: a scoping review. SN Soc Sci.

[48] Arksey H, O'Malley L (2005). Scoping studies: towards a methodological framework. Int J Soc Res Methodol.

[49] Tricco AC, Lillie E, Zarin W, O'Brien KK, Colquhoun H, Levac D (2018). PRISMA extension for scoping reviews (PRISMA-ScR): checklist and explanation. Ann Internal Med.

[50] Wadi M, Yusoff MSB, Abdul Rahim AF, Lah NAZN (2022). Factors affecting test anxiety: a qualitative analysis of medical students’ views. BMC Psychol.

[51] Fowell S, Southgate L, Bligh J (1999). Evaluating assessment: the missing link?. Med Educ.

[52] Lineberry M. Assessment affecting learning. Assessment in health professions education. 2nd ed. New York: Routledge Taylor and Francis Group; 2019. p. 257–71.

[53] Lynn MR (1986). Determination and quantification of content validity. Nurs Res.

[54] Yusoff MSB (2019). ABC of content validation and content validity index calculation. Res.

[55] Grant JS, Davis LL (1997). Selection and use of content experts for instrument development. Res Nurs Health.

[56] Polit DF, Beck CT (2006). The content validity index: are you sure you know what's being reported? Critique and recommendations. Res Nurs Health.

[57] Wolfe DA, Stasny EA. Encyclopedia of Survey Research Methods. 2008 2022/07/04. Thousand Oaks Thousand Oaks, California: Sage Publications, Inc. Available from: https://methods.sagepub.com/reference/encyclopedia-of-survey-research-methods.

[58] Cook DA, Beckman TJ (2006). Current concepts in validity and reliability for psychometric instruments: theory and application. Am J Med.

[59] Yusoff MSB (2019). ABC of response process validation and face validity index calculation. Educ Med J..

[60] Martin AJ, Marsh HW (2006). Academic resilience and its psychological and educational correlates: a construct validity approach. Psychol Schools.

[61] Dunn LB, Iglewicz A, Moutier C (2008). A conceptual model of medical student well-being: promoting resilience and preventing burnout. Acad Psychiatry.

[62] Martin AJ, Marsh HW (2008). Academic buoyancy: towards an understanding of students' everyday academic resilience. J School Psychol.

[63] Kunicki ZJ, Harlow LL (2020). Towards a higher-order model of resilience. Soc Indicat Res.

[64] Van Der Vleuten CPM (1996). The assessment of professional competence: developments, research and practical implications. Adv Health Sci Educ.

[65] Gibbs G, Simpson C, Macdonald R, editors. Improving student learning through changing assessment–a conceptual and practical framework. European Association for Research into Learning and Instruction Conference, Padova, Italy; 2003: Citeseer. (Retrieved from: https://citeseerx.ist.psu.edu/document?repid=rep1&type=pdf&doi=ca609b98befc83caf868ca6c28226cc8acc44d51).

[66] Baartman LK, Bastiaens TJ, Kirschner PA, Van der Vleuten CP (2006). The wheel of competency assessment: presenting quality criteria for competency assessment programs. Stud Educ Eval.

[67] Nicol DJ, Macfarlane-Dick D (2006). Formative assessment and self-regulated learning: a model and seven principles of good feedback practice. Stud Higher Educ.

[68] Dijkstra J, Van der Vleuten C, Schuwirth L (2010). A new framework for designing programmes of assessment. Adv Health Sci Educ.

[69] Norcini J, Anderson B, Bollela V, Burch V, Costa MJ, Duvivier R (2011). Criteria for good assessment: consensus statement and recommendations from the Ottawa 2010 conference. Med Teach.

[70] Cilliers FJ, Schuwirth LWT, van der Vleuten CPM (2012). Modelling the pre-assessment learning effects of assessment: evidence in the validity chain. Med Educ.

[71] ASPIRE. Aspire recognition of excellence in assessment in a medical school. Available on: http://www.aspire-to-excellence.org/Areas+of+Excellence/.2013.

[72] Sarason IG, Sarason IG (1980). Introduction to the study of test anxiety. Test anxiety: Theory, research, and applications.

[73] Naveh-Benjamin M, McKeachie WJ, Lin YG, Holinger DP (1981). Test anxiety: Deficits in information processing. J Educ Psychol..

[74] Hodapp V, Henneberger A. Test anxiety, study habits, and academic performance. In: Spielberger CD, van der Ploeg HM, Schwarzer R, editors. Advances in test anxiety research. 2. Lisse, the Netherlands: Swets and Zeitlinger; 1983. p. 119–27. (Retrieved from:https://www.researchgate.net/profile/Wim-Kleijn/publication/15307613_Cognition_Study_Habits_Test_Anxiety_and_Academic_Performance/links/56f06bd008ae70bdd6c94b77/Cognition-Study-Habits-Test-Anxiety-and-Academic-Performance.pdf).

[75] Smith RJ, Arnkoff DB, Wright TL (1990). Test anxiety and academic competence: A comparison of alternative models. J Counsel Psychol.

[76] Carver CS, Scheier MF (1990). Origins and functions of positive and negative affect: A control-process view. Psychol Rev.

[77] Covington MV. Making the grade: A self-worth perspective on motivation and school reform. New York, NY, US: Cambridge University Press; 1992. 10.1017/CBO9781139173582.

[78] Spielberger CD, Vagg PR (1995). Test anxiety: Theory, assessment, and treatment.

[79] Sansgiry SK (2006). Effect of students' perceptions of course load on test anxiety. Am J Pharma Educ.

[80] Sansgiry S, Bhosle M, Dutta AP (2005). Predictiors of test anxiety in doctor of pharmacy students: an empirical study. Pharm Educ.

[81] Zhang N, Henderson CN (2019). Predicting stress and test anxiety among 1st-year chiropractic students. J Chiropractic Educ.

[82] Edelman M, Ficorelli C (2005). A measure of success: nursing students and test anxiety. J Nurs Staff Dev.

[83] Loya NS, Jiwane NN. Exam anxiety in professional medical students. Int J Res Rev. 2019. https://mail.jpma.org.pk/PdfDownload/1364

[84] Son HK, So W-Y, Kim M (2019). Effects of aromatherapy combined with music therapy on anxiety, stress, and fundamental nursing skills in nursing students: a randomized controlled trial. Int J Environ Res Public Health.

[85] Brodersen LD (2017). Interventions for test anxiety in undergraduate nursing students: an integrative review. Nurs Educ Perspect.

[86] Shapiro AL (2014). Test anxiety among nursing students: a systematic review. Teach Learn Nurs.

[87] Johnson CE (2014). Effect of aromatherapy on cognitive test anxiety among nursing students. Altern Complement Ther.

[88] Furlong E, Fox P, Lavin M, Collins R (2005). Oncology nursing students' views of a modified OSCE. Eur J Oncol Nurs : J Eur Oncol Nurs Soc.

[89] Young I, Montgomery K, Kearns P, Hayward S, Mellanby E (2014). The benefits of a peer-assisted mock OSCE. Clin Teach.

[90] Zhang N, Walton DM (2018). Why so stressed? A descriptive thematic analysis of physical therapy students' descriptions of causes of anxiety during objective structured clinical exams. Physiother Can.

[91] Panadero E, Broadbent J, Boud D, Lodge JM (2019). Using formative assessment to influence self- and co-regulated learning: the role of evaluative judgement. Eur J Psychol Educ.

[92] Panadero E, Andrade H, Brookhart S (2018). Fusing self-regulated learning and formative assessment: a roadmap of where we are, how we got here, and where we are going. Aust Educ Res.

[93] Morris R, Perry T, Wardle L (2021). Formative assessment and feedback for learning in higher education: a systematic review. Rev Educ.

[94] Powell DE, Carraccio C (2018). Toward competency-based medical education. New England J Med.

[95] Van Melle E, Frank JR, Holmboe ES, Dagnone D, Stockley D, Sherbino J (2019). A core components framework for evaluating implementation of competency-based medical education programs. Acad Med.

[96] Schuwirth LWT, van der Vleuten CPM (2020). A history of assessment in medical education. Adv Health Sci Educ.

[97] To J, Panadero E, Carless D (2021). A systematic review of the educational uses and effects of exemplars. Assess Eval Higher Educ.

[98] Rust C, Price M, O'Donovan B (2003). Improving students' learning by developing their understanding of assessment criteria and processes. Assess Eval Higher Educ.

[99] Nicol D (2021). The power of internal feedback: exploiting natural comparison processes. AssessEval Higher Educ.

[100] De La Fuente J, López-García M, Mariano-Vera M, Martínez-Vicente JM, Zapata L (2017). Personal self-regulation, learning approaches, resilience and test anxiety in psychology students. Estud Sobre Educ.

[101] Cipra C, Müller-Hilke B (2019). Testing anxiety in undergraduate medical students and its correlation with different learning approaches. PLoS ONE.

[102] Ahmady S, Khajeali N, Kalantarion M, Sharifi F, Yaseri M (2021). Relation between stress, time management, and academic achievement in preclinical medical education: a systematic review and meta-analysis. J Educ Health Promot.

[103] McManus IC, Thompson M, Mollon J (2006). Assessment of examiner leniency and stringency ('hawk-dove effect') in the MRCP(UK) clinical examination (PACES) using multi-facet Rasch modelling. BMC Med Educ.

[104] McKevitt CT (2016). Engaging students with self-assessment and tutor feedback to improve performance and support assessment capacity. J Univ Teach Learn Pract.

[105] Bing-You R, Hayes V, Varaklis K, Trowbridge R, Kemp H, McKelvy D (2017). Feedback for learners in medical education: what is known? A scoping review. Acad Med..

[106] van der Vleuten CPM, Schuwirth LWT, Driessen EW, Dijkstra J, Tigelaar D, Baartman LKJ (2012). A model for programmatic assessment fit for purpose. Med Teach.

[107] Branch WT, Paranjape A (2002). Feedback and reflection teaching methods for clinical settings. Acad Med..

[108] Veine S, Anderson MK, Andersen NH, Espenes TC, Søyland TB, Wallin P (2020). Reflection as a core student learning activity in higher education - Insights from nearly two decades of academic development. Int J Acad Dev.

[109] Stern C, Kleijnen J (2020). Language bias in systematic reviews: you only get out what you put in. JBI Evid Synth.

[110] Neimann Rasmussen L, Montgomery P (2018). The prevalence of and factors associated with inclusion of non-English language studies in Campbell systematic reviews: a survey and meta-epidemiological study. Syst Rev.

