# Supplementary material for: The framework of Systematic Assessment for Resilience (SAR): development and validation
Source: BMC Med Educ. 2023 Apr 5;23:213. doi: 10.1186/s12909-023-04177-5 (PMC10073620; doi:10.1186/s12909-023-04177-5)
Supplement: Supplementary file 1 — Additional file 1: Appendix I. (Three narrative reviews). Table S1. The identified theoretical frameworks of academic resilience. Table S2. The identified theoretical frameworks of assessment. Table S3. The identified theoretical frameworks of test anxiety. Appendix II. (Scoping review). Figure S1. PRISMA chart of the scoping review identifying factors related to test anxiety. Table S3. Descriptive variables of the included studies (n = 74). Table S4. Descriptive variables of the included studies (n = 74). Table S5. Themes of factors that increase test anxiety. Table S6. Themes of factors that decrease test anxiety. Appendix III. (Focus Group Discussion). Figure S2. Emerged themes and sub-themes in relation to increasing and decreasing test anxiety. Table S7. Emerged themes and sub-themes from FGD with supporting quotations. Appendix IV. (Generating resilience guidelines based on the scoping review and FGD ). Figure S3. Relation of the proposed guidelines with outputs of scoping review and FGD. Appendix V. (Content Validation). Table S8. Content Validation Index - Guidelines rated as 3 or 4 (relevant) is ticked on the table. Table S9. Acceptable values for content validity indices (104). Table S10. Demographic data of the expert panels. Table S11. The original version of the SAR guidelines that was sent for content validation. Appendix VI. (Response Process). Table S12. Content Validation Index - Guidelines rated as 3 or 4 (relevant) is ticked on the Table S. Table S13. Demographic data of the panels in face validation study. Table S14. The FVI indices of the 19 SAR guidelines. Appendix VII. (Written response of medical teacher in using SAR guideline). Table S15. The medical teachers’ feedback after response process. [file 12909_2023_4177_MOESM1_ESM.docx]

**Supplementary files (appendices)**

Table of Contents

[Appendix I: (Three narrative reviews) 2](#_Toc108978917)

[Appendix II: (Scoping review) 10](#_Toc108978918)

[Appendix III: (Focus Group Discussion) 54](#_Toc108978919)

[Appendix IV: (Generating resilience guidelines based on the scoping review and FGD ) 58](#_Toc108978920)

[Appendix V: (Content Validation) 59](#_Toc108978921)

[Appendix VI: (Response Process) 63](#_Toc108978922)

[Appendix VII: (Written response of medical teacher in using SAR guideline) 66](#_Toc108978923)

# **Appendix I: (Three narrative reviews)**

**General information:**

**Aim:**

These three narrative reviews explored the previously published articles that discussed theories/frameworks and their underlying principles and application of three areas: academic resilience, test anxiety, and assessment system.

**Searching data bases:**

The researcher conducted the search in four databases: PubMed, CINAHL, PsycINFO and Google Scholar. The used keywords depend on the previously mentioned four concepts. The Boolean term “AND” with limitation in publication date between 1990 till 2020 was performed.

Eligibility criteria:

Articles were screened and selected based on the following eligibility criteria:

1. Original article
2. Clearly describing the development/construction of a theoretical foundation in the selected areas of research.
3. Written in English

**Data synthesis:**

The selected articles were compiled in an evidence synthesis Table S1 to extract key information about the theoretical foundations of psychological resilience, academic resilience, test anxiety, and assessment system. The following sub-sections showed the results.

**1^st^ Narrative review: Academic Resilience:**

Regarding the narrative review concerning “academic resilience”, the initial search resulted in the retrieval of 164 articles from four databases. Further filtration by relevant title and abstract reading yields five articles that meet the eligibility criteria. Table S1 showed a summary of the included articles.

Table S1: The identified theoretical frameworks of academic resilience.

| **Author(s), year** | **Study title** | **Main study purpose** | **The name of the theory/theoretical foundation/framework** | **Description** |
| --- | --- | --- | --- | --- |
| Martin (1) | Motivation and Academic Resilience: Developing a Model for Student Enhancement | To create a model that will assist teachers in encouraging academic resilience in their students. | Motivation boosters and guzzlers model | This model explains how to promote motivation, as a precursor of resilience, in an educational setting. Martin (1) developed his model by synthesizing various prior theories. The model is predicated on increasing motivation through "boosters" and decreasing its adversarial "guzzlers."  **Motivation boosters:**   - Self-belief - Value of schooling - learning focus, persistence, planning and monitoring, and study management   **Motivation guzzlers:**   - Low control - Avoidance, self-sabotage, and anxiety |
|  |  |  |  |  |
| Martin and Marsh (2) | Academic resilience and its psychological and educational correlates: A construct validity approach | To explore an academic resilience model through a path analysis method | The 5-C (confidence, coordination, control, composure and commitment) model of academic resilience. | The author proposed five constructs that shape academic resilience in this model (2). These included the following:   - Confidence, is synonymous with self-efficacy - Coordination, which means the ability of planning study tasks - Control, which refers to the ability to exert control over perceived uncertainty. - Composure, which indicates a low level of anxiety - Commitment, which entails keeping persistence. |
|  |  |  |  |  |
| Dunn, Iglewicz (3) | A conceptual model of medical student well-being: promoting resilience and preventing burnout | To propose a novel conceptual model, the “coping reservoir,” of medical student wellbeing. | Coping reservoir tank | This framework represents a hypothetical tank, which corresponds to one's mental state, and describes the circumstances that result in its depletion and replenishment (3). The following factors contribute to its depletion:   - Stress - Internal Conflict - Time and Energy Demands   While those who replenish it are as follows:   - Psychosocial Support - Social/healthy Activities - Mentorship - Intellectual Stimulation |
|  |  |  |  |  |
| Martin and Marsh (4) | Academic buoyancy: Towards an understanding of students' everyday academic resilience | To investigate everyday academic buoyancy and resilience and correlate the results with theoretical foundation. | Academic buoyancy process model. | Martin and Marsh (4) argued in this model that academic buoyancy is distinct from academic resilience and that promoting daily buoyancy automatically increases academic resilience. As a result, the authors proposed this model, describing how the following factors contribute to buoyancy and, ultimately, resilience:   - Increase self-efficacy - More engagement in the academic context - Low anxiety environment - More ability to control surroundings - Good teacher-students relationship |
|  |  |  |  |  |
| Kunicki and Harlow (5) | Towards a Higher‐Order Model of Resilience | The current study developed and tested a higher-order model of resilience with under- lying components from a trait resilience perspective | Higher-order model of resilience | According to Kunicki and Harlow (5), six constructs interact to promote higher-order resilience. These factors are:   - purpose in life, - self-esteem, - life satisfaction, - cognitive flexibility, - proactive coping, and - social support. |

**2^nd^ Narrative review: student assessment**

This narrative review summarizes the existing assessment frameworks, describes their underlying principles, and identifies areas in which resilience can be promoted. Eight articles out of 96 met the criteria. Table S2 shows brief description of these articles.

Table S2: The identified theoretical frameworks of assessment.

| **Author(s), year** | **Study title** | **Main study purpose** | **The name of the theory/theoretical foundation/framework** | **Description** |
| --- | --- | --- | --- | --- |
| Van Der Vleuten (6) | The assessment of professional competence: Developments, research and practical implications | Evaluating the main outcomes of assessment research in HPE and suggesting a theoretical framework | Utility of assessment = validity (V)🞨reliability (R)🞨educational impact (E) 🞨acceptability (A) 🞨cost (C) | Van Der Vleuten (6) argued that assessment should adhere to all components of this hypothetical formula, and that any violation will raise doubts about the assessment's robustness. Since its inception, assessment utility has been widely used as a foundation for other frameworks. The formula considers five assessment pillars: validity (V), reliability (R), educational impact (E), acceptability (A), and cost (C). |
|  |  |  |  |  |
| Gibbs, Simpson (7) | Improving student learning through changing assessment–a conceptual and practical framework | To develop a conceptual framework to support student learning under assessment | The supporting framework for student learning under assessment | Based on theory and empirical evidence, Gibbs, Simpson (7) proposed an eleven-point list of "conditions under which assessment most effectively supports learning." These conditions apply to how assessments collect and distribute students' time and effort throughout a course, how assessments generate productive learning activity, how assessments provide adequate prompt feedback, feedback quality, and how students respond to and use feedback to inform subsequent learning. This is the list:  **“Quantity and distribution of student effort**  1. Assessed tasks capture sufficient study time and effort  2. These tasks distribute student effort evenly across topics and weeks  **Quality and level of student effort**  3. These tasks engage students in productive learning activity  4. Assessment communicates clear and high expectations to students  **Quantity and timing of feedback**  5. Sufficient feedback is provided, both often enough and in enough detail  6. The feedback is provided quickly enough to be useful to students  **Quality of feedback**  7. Feedback focuses on learning rather than on marks or students themselves  8. Feedback is linked to the purpose of the assignment and to criteria  9. Feedback is understandable to students, given their sophistication  **Student response to feedback**  10. Feedback is received by students and attended to  11. Feedback is acted upon by students to improve their work or their learning”. |
|  |  |  |  |  |
| Baartman, Bastiaens (8) | The wheel of competency assessment: Presenting quality criteria for competency assessment programs | To combine different methods of competency assessment under one framework. | The wheel of competency assessment program | The Wheel of Competence Assessment Programs was developed by Baartman et al. (8). It is a self-assessment tool for determining the efficacy of assessment programmes. It includes 1) acceptability, 2) authenticity, 3) cognitive complexity, 4) comparability, 5) cost and efficiency, 6) educational consequences, 7) fairness, 8) fitness for purpose, 9) fitness for self-assessment, 10) meaningfulness, 11) reproducibility of decisions and 12) transparency. |
|  |  |  |  |  |
| Nicol and Macfarlane‐Dick (9) | Formative assessment and self‐regulated learning: A model and seven principles of good feedback practice | In relation to the development of self-regulation, the authors propose seven principles of good feedback practice. | Good feedback practice model | The authors reinterpreted existing research on formative assessment and feedback in this model (9) and demonstrated how these processes can assist students in taking control of their own learning, becoming self-regulated learners. They proposed seven self-regulation-supporting principles of good feedback practice, where were:   1. helps clarify what good performance is (goals, criteria, expected standards); 2. facilitates the development of self-assessment (reflection) in learning; 3. delivers high quality information to students about their learning; 4. encourages teacher and peer dialogue around learning; 5. encourages positive motivational beliefs and self-esteem; 6. provides opportunities to close the gap between current and desired performance; 7. provides information to teachers that can be used to help shape teaching”. |
|  |  |  |  |  |
| Dijkstra, Van der Vleuten (10) | A new framework for designing programmes of assessment | To create a model for framing assessment programmes and determining which dimensions should be considered when formulating design criteria | A new model for designing programs of assessment | Dijkstra, Van der Vleuten (10) developed a new model for developing assessment programmes that takes a holistic view of all assessment elements rather than individual components. He later published a 72-item guideline for assessment programmes (11). |
|  |  |  |  |  |
| Norcini, Anderson (12) | Criteria for good assessment: consensus statement and recommendations from the Ottawa 2010 Conference | To come up with criteria of good assessment | Criteria of good assessment | A group of educational measurement experts developed a consensus statement and recommendations outlining the criteria for good assessment during the Ottawa 2010 Conference (12). The criteria are a collection of assessment principles that include the following: (1) validity or coherence, (2) reproducibility or consistency, (3) equivalence, (4) feasibility, (5) educational effect, (6) catalytic effect, and (7) acceptability. |
|  |  |  |  |  |
| Cilliers, Schuwirth (13) | A model of the pre-assessment learning effects of summative assessment in medical education | To configure a model illustrating the effects of assessment (summative) on student learning | A model of the pre-assessment learning effects on student assessment. | Cilliers, Schuwirth (13)proposed that assessment has an impact on student learning through two mechanisms: task demands and system design. They operated through four facets of the mechanism, namely impact appraisal, response appraisal, perceived agency, and interpersonal factors, to produce two pre-assessment learning effects: the nature of cognitive processing activities and metacognition regulation activities. |
|  |  |  |  |  |
| ASPIRE (14) | Aspire recognition of excellence in assessment in a medical school | To set criteria for excellence in assessment practice in medical schools. | Aspire criteria for assessment | ASPIRE is an initiative by the Association for Medical Education in Europe (AMEE) launched in 2013 (15). This initiative aims to promote excellence in certain fields of medical education and to allow schools to be recognized internationally for their excellence in those are. The criteria for excellence in assessment include: “1) the assessment program serves and supports the mission of the institution and the goal of medical education globally in enhancing and improving the health of both populations and individuals. 2) the assessment program supports, enhances and creates learning opportunities. 3) the assessment program ensures the competence of students as they progress. 4) the assessment program is subject to a rigorous and continuous quality control process. 5) the assessment program demonstrates a commitment to continuous scholarship and innovation. For each criterion, assessors seek evidence to approve their implementation”. |

**3^rd^ Narrative review: Test anxiety:**

There were several theories describing test anxiety and its underlying mechanisms. This narrative review, the researcher found seven studies met the selection criteria. Table S3 summarizes and describes these theories.

Table S3: The identified theoretical frameworks of test anxiety.

| **Author(s), year** | **Study title** | **Theories/theoretical foundations/framework** | **Description** |
| --- | --- | --- | --- |
| Naveh-Benjamin, McKeachie (16), Sarason (17) | Test anxiety: Deficits in information processing. | The cognitive attentional model | In this model, Naveh-Benjamin, McKeachie (16) proposed that there are two groups of variables interfere with test performance; cognitive and emotional reactions. In cognitive group, students have negative evaluation and self-perception of inadequate competence, while in the second group, emotionality, students have been experienced with physiological reaction on the test day. |
|  |  |  |  |
| Hodapp and Henneberger (18) | Test anxiety, study habits, and academic performance | The learning deficit model | This model establishes a connection between the situation of insufficient exam preparation and the occurrence of test anxiety (18). |
|  |  |  |  |
| Smith, Arnkoff (19) | Test anxiety and academic competence: A comparison of alternative models | The dual deficit model | The cognitive attentional model and the learning deficit model are combined in this model, which includes all of the characteristics of both models. Smith, Arnkoff (19) proposed that test anxiety is a result of the interaction of all of these factors. |
|  |  |  |  |
| Carver and Scheier (20) | Origins and functions of positive and negative affect: A control-process view | Self-regulation model | In this model, Carver and Scheier (20) predicated on the premise that human intentional goal-directed behavior exhibits functional characteristics of a feedback control system. As a result, individuals establish personal goals and standards that serve as reference points for guiding and monitoring their behavior. Current behaviors are constantly sensed and recalled, and then compared to contextually relevant reference values and goals. Discrepancies between observed behaviors or states and significant reference values or behavioral standards are resolved by adjusting behavior in the direction of the latter. Accordingly, it may result in test anxiety. |
|  |  |  |  |
| Covington (21) | Making the grade: A self-worth perspective on motivation and school reform | Self-Worth Model | According to the self-worth model (21), test anxiety is frequently best understood in terms of an individual's efforts to maintain self-worth and a positive self-image, especially when confronted with the prospect of academic failure. |
|  |  |  |  |
| Spielberger and Vagg (22) | Test anxiety: Theory, assessment, and treatment | Transactional Process Model | This model emphasizes the dynamic interaction and mutual determinism of the various components of the stress process: individuals, situations, affective reactions, coping behaviors, and adaptive outcomes. This model emphasizes the interaction of personality characteristics and environmental stressors in determining anxiety states and the critical role of cognitive appraisals as mediating factors between people and situations in affecting state anxiety (22). |

# **Appendix II: (Scoping review)**

**General information:**

**Aim:**

The scoping review is focused on identification of factors influencing test anxiety. Key terms were developed to capture literature that related to test anxiety and its related factors. The time period 1990 to 2020 was considered in the scoping review.

**Searching data bases:**

The following SIX electronic databases were searched: PubMed, CINAHL, PsychINFO, and ERIC (through EBSCOHST), SCOPUS, and ProQuest.

**Data extraction:**

Guided by the inclusion and exclusion criteria, articles were screened by their titles and abstracts. The reporting of article selection followed the Preferred Reporting of Items for Systematic Reviews and Meta- Analyses (PRISMA) Statement (23). Key information from the included articles were charted onto a form that is developed based on the purpose of the scoping review.

**Data synthesis:**

Based on the factors identified in each study, related codes from all studies were collated to generate subthemes and overarching themes. The major themes, which were composed of frequently reported and overlapping factors, were chosen, renamed, and included in the final analysis.

**Identification**

Additional citations identified through other sources (n= 38)

Titles and abstracts identified through database searching (n = 957)

**Screening**

**Eligibility**

**Included**

661 titles and abstracts were excluded on basis of duplication

Titles and abstracts screened after duplicates removed

**(n = 995)**

171 abstracts were excluded:

- 121 studies are not HPE
- 50 studies are school’s students

Abstracts screened based on eligibility criteria

**(n= 334)**

89 articles were excluded:

- 79 not meet eligibility criteria
- 7 not accessed
- 2 Non-English

Full- text articles screened **(n=163)**

Studies included in the Scoping Review

**(n = 74)**

Figure S1: PRISMA chart of the scoping review identifying factors related to test anxiety

Table S3: Descriptive variables of the included studies (n = 74)

| Variables | Detail | N | % |
| --- | --- | --- | --- |
| Years category | 1990 - 2000 | 2 | 3% |
|  | 2001 - 2010 | 12 | 16% |
|  | 2011- 2020 | 60 | 81% |
|  |  |  |  |
| Country | USA | 27 | 36% |
|  | Iran | 6 | 8% |
|  | India | 5 | 7% |
|  | UK | 4 | 5% |
|  | Canada | 4 | 5% |
|  | Saudi Arabia | 4 | 5% |
|  | Pakistan | 3 | 4% |
|  | Australia | 2 | 3% |
|  | South Korea | 2 | 3% |
|  | Germany | 2 | 3% |
|  | New Zealand | 2 | 3% |
|  | Ireland | 1 | 1% |
|  | Netherlands | 1 | 1% |
|  | Ireland | 1 | 1% |
|  | Malaysia | 1 | 1% |
|  | Norway | 1 | 1% |
|  | Italy | 1 | 1% |
|  | Romania | 1 | 1% |
|  | Turkey | 1 | 1% |
|  | Spain | 1 | 1% |
|  | China | 1 | 1% |
|  | Belgium | 1 | 1% |
|  | Ethiopia | 1 | 1% |
|  | Israel | 1 | 1% |
|  |  |  |  |
| Study design | Cross-sectional study | 35 | 47% |
|  | Experimental study | 14 | 19% |
|  | Review study | 8 | 11% |
|  | Mixed study design | 9 | 12% |
|  | Qualitative study | 4 | 5% |
|  | Prospective study | 4 | 5% |
|  |  |  |  |
| Students category | Medical students | 28 | 38% |
|  | Nursing students | 21 | 28% |
|  | Dental students | 8 | 11% |
|  | Pharmacy students | 3 | 4% |
|  | Physiotherapy students | 3 | 4% |
|  | Mix of health professions students | 6 | 8% |
|  | Veterinary students | 1 | 1% |
|  | Occupational therapy | 1 | 1% |
|  | Radiology students (sonographic) | 1 | 1% |
|  | Public health | 1 | 1% |
|  | Chiropractic | 1 | 1% |

Table S4: Descriptive variables of the included studies (n = 74)

| Authors (year) | Country/  location | Target students | Purpose | Method/Sample/comparison group | Main study findings: | Factors that increase TA | Factors that decrease TA |
| --- | --- | --- | --- | --- | --- | --- | --- |
| Rukholm and Viverais (24) | Canada | Nurse students | The goal is to determine anxiety levels and coping strategies of registered nurse students from on-campus and distance education at three different points during the challenge examination process. | - Prospective study - 105 nurse students, who undertook the "challenge examination" - Anxiety levels and coping methods were measured repeatedly over time. | Anxiety levels, coping strategies, and past academic performance were found to differ significantly by age, marital status, employment, and work setting. A variety of coping strategies were employed. However, both groups used problem solving extensively, while on-campus students used social support more frequently. | - Young age - Poor past academic performance - Being single - Unemployed | - Adult age - Good past academic performance - Being married - Employed - Confrontive coping - Self-controlling - Seeking social support - Escape - avoidance - Planful problem solving |
| Malathi and Damodaran (25) | India | Medical students | The aim is to see if yogic practices have any effect on anxiety levels during daily activities and prior to examination. | - Experimental study (pre and post) - 50 students of first year MBBS program. - They were randomly divided into two groups; control and yoga groups | Following yoga practice, Spillberger's anxiety scale showed a statistically significant decrease. Furthermore, after practice, the anxiety score, which had risen prior to exams, showed a statistically significant reduction on the day of the exam. |  | - Yoga |
| Broyles, Cyr (26) | USA | Medical students | The goal is to see if students benefit from being able to consult the textbook during the exam in order to get the most educational value out of the final clerkship exam. | - Mixed study design (experimental + qualitative study) - There is no control group | When comparing students who took an open-book approach to those who took a closed-book approach, there was a statistically significant difference in mean score. Clearly, the procedure reduced anxiety and created a secure environment for evaluation. |  | - Open book test |
| Edelman and Ficorelli (27) | UK | Nursing students | The study aimed to give an insight into the lives of nursing students who struggle with test anxiety. | - Qualitative study (unstructured interview) - Eight female students were chosen. - Thematization analysis was done. | Three themes emerged: (a) the reality of an anxiety attack; (b) the academic consequences of test anxiety; and (c) effective anxiety management strategies. Under the column titled "Factors Contributing to Test Anxiety Reduction," techniques contributing to test anxiety reduction were identified and summarized. |  | - Guided imagery - Diaphragmatic breathing, - Muscle relaxation - Note taking - Comprehension reading - Read the rationales for the correct/incorrect answers - Group study sessions - Scheduled periods |
| Furlong, Fox (28) | Ireland | Nursing students | To ascertain students' perceptions of their preparation for the examination, their level of stress and anxiety, and finally, students' perceptions of the OSCE's efficacy in testing clinical skills and the relevance of the skills tested. | - Cross-sectional study - 185 oncology nursing students participated in this study. - A questionnaire was used with 5-point Likert scale to measure students’ perception of an OSCE. | Despite their extensive preparation and positive attitudes toward the relevance of the skills tested, nearly 90% of students rated the OSCE as stressful. | - OSCE |  |
| Sansgiry, Bhosle (29) | USA | Pharmacy students | The purpose of this study was to look into the factors that influence test anxiety in Doctor of Pharmacy students in their first three didactic years at two different universities. | - Cross-sectional study - A scale that measured test anxiety was adapted from a previously validated test-anxiety inventory. - 244 students participated | Reduced test anxiety requires study strategies, time management, test competitiveness, and academic competence. Most importantly, among pharmacy students, test competence and academic competence were significant predictors of test anxiety. |  | - Study strategies - Time management - Test competitive - Academic competence |
| Rohe, Barrier (30) | USA | Medical students | The study aimed to assess the impact of a pass-fail grading system on medical students' stress, mood, group cohesion, and test anxiety. | - Prospective study - Students in the previous class of 2005 (n = 41), who were graded using a 5-interval system during their first year of medical school, were compared with students in the class of 2006 (n = 40). - Perceived Stress Scale, Profile of Mood States, Perceived Cohesion Scale, Test Anxiety Inventory were used. | Students who were graded on a pass-fail system reported less stress than their peers who were graded on a 5-interval system. The mood was better in the pass-fail group, but the difference was not statistically significant (P=.07). There were no significant differences in test-taking anxiety or United States Medical Licensing Examination Step 1 board scores between the two groups. |  | - Pass-fail grading system |
| Sansgiry and Sail (31) | USA | Pharmacy students | The goal of this study is to see if there's a link between student perceptions of course load, time management ability, and test anxiety. | - Cross-sectional study - 198 students participated in the survey - A survey with items measuring test anxiety, perceived course load, and ability to manage time was used. | Students' perceptions of course load were positively related to test anxiety, but their ability to manage time with course work was negatively related. | - Course load | - Good time management |
| Stewart, Hauge (32) | USA | Medical students | The goal of the study was to see how a pre-clinical skills course affected students' ratings of proficiency, confidence, and anxiety. | - Cross-sectional study. - Prior to the CRASH course, 118 students completed a self-assessment of their proficiency, confidence, and level of anxiety in performing these procedural skills, regardless of whether or not they participated. | There was a significant improvement in self-assessed proficiency, confidence, and anxiety after the course. |  | - CRASH course |
| Hashmat, Hashmat (33) | Pakistan | Medical students | The purpose of this study was to use the VAS (Visual Analogue Scale) to assess exam-related anxiety in final professional medical students and to determine the factors that contribute to exam anxiety in final professional medical students. | - Cross-sectional study - 200 medical students were participated. - Exam Anxiety was measured using Visual Analogue Scale (VAS). | Extensive course loads (90.8 %), lack of physical exercise (90 %), and long exam duration (77.5 %) were the most important factors reported by students as contributing to exam anxiety. The majority of students were unaware of exam-taking and anxiety-reduction techniques, and those who were aware of them did not use them. | - Females - Extensive course load - Long duration of exams - Lack of knowledge regarding exam taking - Lack of knowledge regarding anxiety reduction techniques |  |
| Pahwa, Goyal (34) | India | Medical students | The goal of the study was to identify personality traits in medical students and to see if there was a link between pre-exam anxiety and vulnerable personality traits. | - Cross-sectional study - 91 medical students were participated. - The Eysenck Personality Questionnaire and Beck's Anxiety Inventory were used to determine if there was a dominant personality trait. | Medical students, particularly females, experience a significant increase in anxiety before exams. There is a growing trend of neuroticism and extraversion personality types being linked. | - Females - Neuroticism personality - Extraversion personality |  |
| Bloodgood, Short (35) | USA | Medical students | The goal of this study is to see how changing the grading system from graded (A, B, C, D, F) to pass/fail affects medical students' academic performance, attendance, residency match, satisfaction, and psychological well-being in the first two years of medical school. | - Prospective study - Both groups of the graded (n = 141) and pass/fail classes (n = 140) were compared - Web survey (Dupuy General Well-Being Schedule) was administered | During each of the first three semesters of medical school, the pass/fail class showed a significant increase in well-being compared to the graded class. Furthermore, during the first four semesters of medical school, students expressed higher levels of satisfaction with the quality of their medical education, as well as greater satisfaction with their personal lives. |  | - Pass-fail grading system |
| Brand and Schoonheim-Klein (36) | Netherlands | Dental students | The goal of the study was to assess the levels of anxiety, self-perception of preparation, and expectation of success induced by an objective structured clinical examination (OSCE), a written examination, and a preclinical preparation test, as well as to look at the effects of the three predictive variables on the assessments' outcomes. | - Cross-sectional study - Sample size was not clear - There is no control group - Spielberger’s state anxiety inventory | The OSCE is the most common type of assessment that demonstrates anxiety, which is linked to preparation. The level of anxiety experienced during a written examination was linked to the results, but not to preparation. During the preclinical study, state anxiety was found to be linked to preparation and expectation of success. | - OSCE - Time preparation for an exam |  |
| Dayalan, Subramanian (37) | India | Medical students | The study aimed to determine the impact of Mind Sound Technology (MST), an intelligence-enhancing program, on medical undergraduates' psychological well-being during exam stress. | - Experimental study (pre and post) - 42 medical students were recruited and randomized into two groups: non-practitioners and MST practitioners. - Dukes Health Profile scoring was used. | The use of mind sound technology (MST) has helped medical undergraduates cope with stress during exams and improve their academic performance. |  | - Mind Sound Technology (MST) |
| Spring, Robillard (38) | USA | Medical students | The goal of the study was to see how pass/fail grading affected medical students' well-being and academic outcomes. | - Review study (literature review) | A pass/fail evaluation system improves student well-being while having no negative impact on objective academic performance. |  | - Pass-fail grading system |
| O'Carroll and Fisher (39) | UK | Medical students | The goal of this study was to see if the self-regulatory executive functioning (S-REF) model could be used to predict performance test anxiety (PTA) in objective structured clinical examinations (OSCEs). | - Cross-sectional study - 240 Year 1 medical students were participated. - The Metacognitions Questionnaire-30 (MCQ-30), the Penn State Worry Questionnaire (PSWQ), the Attentional Control Scale (ACS), and the Performance Test Anxiety questionnaire were all used in this study (PTA). | The findings back up the S-REF model's predictions that metacognitive beliefs, trait worry, and attentional control processes are at the root of performance test anxiety's onset and maintenance. |  | - Self-regulatory S-REF - Metacognition believes |
| Weeks and Horan (40) | Australia | Physiotherapy students | The aim of this study is to look at a video-based learning activity for engaging physiotherapy students in preparation for practical exams and to see how they perform. | - Mixed study design - 62 physiotherapy students were participated. - Questionnaire-based surveys and focus groups were used. | Participants rated the video examples as an effective learning activity and found the video resources to be effective in supporting their learning (98 % positive) (96 %). Improved understanding, reduced performance anxiety, and enjoyment emerged as themes from focus group responses. |  | - Video-based learning activity |
| Encandela, Gibson (41) | USA | Medical students | The goal of this study was to see how test anxiety affected second-year students preparing for Step 1. | - Mixed study design - 93 second-year students voluntarily participated. - Westside Test Anxiety Scale was used. - The data was gathered at three points: before a formal Step 1 study period, during the study period, and after the vast majority of students had completed the exam. | Students reported that negative self-talk during exam preparation was one of the causes of test anxiety. Anxiety's effects on emotional well-being, cognitive functioning, and physical well-being were studied. Socializing with others and a variety of cognitive and physical approaches were among the strategies used. | - Negative self-talk | - Socialization - Cognitive strategies - Physical strategies |
| Johnson (42) | USA | Nursing students | The purpose of this study was to see how aromatherapy (Citrus Limon [lemon] essential oil) affected nursing students' cognitive test anxiety. The Cognitive Test Anxiety Survey (CTAS) scores were measured before and after the intervention. | - Experimental study (RCT) - 46 nursing students participated - The students were randomized into two groups, control and experimental. | Between the pretest and post-test, the control group's cognitive test anxiety scores decreased by three points. Students who received aromatherapy, on the other hand, had significantly lower cognitive test anxiety scores than those who did not (P = 0.10). |  | - Aromatherapy (lemon essential oil) |
| Lyndon, Strom (43) | New Zealand | Medical students | The goal of the study was to see if there was a link between academic assessment and psychological distress among medical students so that assessment practices could be improved. | - Systematics review | Assessment causes stress or anxiety, according to the findings, perhaps more so for female medical students. Other factors were also taken into account. | - Female - Clinical exams - Past poor assessment performance | - Open book - Pass-fail grading system |
| Muldoon, Biesty (44) | Ireland | Nursing students | The purpose of this study is to describe how a survey of midwifery students' attitudes toward a Lactation and Infant Feeding OSCE was conducted and the results, as well as to consider these attitudes in light of the international literature and empirical evidence base. | - Cross-sectional study - 35 nursing students participated. - An 18-item Likert scale was used in a descriptive survey design. | Midwifery students were neutral or unsure about the OSCE as a strategy for assessing clinical competence, according to the findings of this study. The OSCE elicits nervousness (mean 4.27) and stress in a significant number of students (mean 4.30). | - OSCE |  |
| Rajiah and Saravanan (45) | Malaysia | Pharmacy students | The goal of the study is to look at the impact of a psychological intervention on reducing performance anxiety in first-year pharmacy students, as well as the consequences of the intervention. | - Experimental study (pre and post) - Initial, 236 first-year undergraduate pharmacy students were participated. - Westside Test Anxiety Scale (WTAS), the Kessler Perceived Distress Scale (PDS), and the Academic Motivation Scale (AMS) were used. - Then, 42 students (with moderate to high anxiety) were randomly assigned to one of two groups: the experiment (n=21) or the waiting list control (n=21). | The anxiety management of psychoeducation and systematic education for test anxiety in this study improved grade point average and reduced lack of motivation and psychological distress (GPA). |  | - Brief psychoeducation - Systematic desensitization - Individual counseling |
| Røykenes, Smith (46) | Norway | Nursing students | The goal of the study was to look into nursing students' test anxiety when they were given a high-stakes drug calculation test. | - Mixed study design - a survey questionnaire and a focus group interview were used. - The survey was completed by 203 nursing students, six of whom also took part in the focus group interview. | In the months prior to the drug calculation test, 44.3 percent of students reported high mathematics test anxiety, according to the survey results. The data from the focus group interviews revealed that the high stakes of the test significantly increased test anxiety. | - High stakes exam |  |
| Shapiro (47) | USA | Nursing students | The goal of the study was to look into the factors that contribute to test anxiety in nursing students. | - Systematic review. | Hypnotherapy, aromatherapy, and relaxation training are just a few of the interventions that have been shown to help with test anxiety symptoms and effects. |  | - Hypnotherapy - Aromatherapy - Relaxation training - Modified cognitive desensitization program - Lento music therapy Test-taking strategy |
| Young, Montgomery (48) | UK | Medical students | The goal of the study was to describe the design and implementation of a peer-run mock OSCE exam for medical students, as well as its feasibility, acceptability, and perceived impact. | - Mixed methods study design - Four fourth-year students created an OSCE training program. - It entailed the hiring of 103 fourth-year tutors to help 245 third-year medical students run OSCE stations and provide feedback prior to their summative end-of-year exam. - A questionnaire was completed by tutees and tutors to assess the quality and perceived benefits of this educational intervention. | The tutees' main themes were increased confidence and appreciated feedback. Motivation to continue with peer-assisted learning (PAL) projects and improved teaching skills were the main themes from the tutor comments. The peer-assisted mock OSCE increased tutee confidence and decreased OSCE-related anxiety. |  | - Peer-assisted mock OSCE |
| Ali, Asim (49) | Pakistan | Medical students | The goal of this study is to see if there's a link between two different systems for evaluating academic performance and stress levels among students at two different medical schools in Karachi, Pakistan. | - Cross-sectional study - 387 medical students participated. - They were from two different systems: semester examinations with grade point average (GPA) scores (a tiered system), and annual examinations with only pass/fail grading. - The Westside Test Anxiety Scale was used to determine test anxiety levels (WTAS). The Perceived Stress Scale was used to assess overall stress (PSS). | The WTAS (2.490.8 vs. 2.890.7; p 0.01) and the PSS (17.096.7 vs. 20.396.8; pB0.01) scores of students in the pass/fail assessment system were lower than those in the GPA assessment system, indicating lower levels of test anxiety and overall stress. Students who used the pass/fail system were happier with their grades than those who used the GPA system. |  | - Pass/fail grading system |
| Chen, Henning (50) | New Zealand | Medical students | The goal of the study was to compare the impact of progress testing on undergraduate medical students' learning styles and stress levels to that of traditional high-stakes exams. | - Quasi-experimental study design - To assess change over time, undergraduate medical students (N = 333 at Time 1 and N = 298 at Time 2) completed the Revised Study Process Questionnaire (R-SPQ-2F) and the Perceived Stress Scale (PSS) at two time points. | Progress testing (PT) appears to have reduced the examination stress of medical students, as students who participated in PT reported lower levels of stress. |  | - Progress testing |
| March and Robinson (51) | USA | Nursing students | The goal of the study was to look into the links between hopeful thinking, goal orientation, and high-stakes testing performance. | - Cross-sectional study. - 151 nursing students participated - Different scales and questionnaires were used. | Performance-avoidance goal scores were significantly related to lower exam scores, while hopeful thinking was significantly related to higher exam scores. |  | - Hopeful thinking - Goal orientation |
| Messineo, Gentile (52) | Italy | Nursing students | In a General Psychology course for undergraduate nursing students, the researchers wanted to see how a test enhanced learning (TEL) program affected long-term retention and how it interacted with students' test anxiety. | - Prospective study - 161 undergraduate nursing students participated. | Test-Enhanced Learning has the potential to be a powerful tool for promoting and improving learning. Indeed, taking tests after studying resulted in better long-term retention and, ultimately, better test performance than re-reading without testing. Students who have a higher level of test anxiety may benefit more from participating in a Test-Enhanced Learning process than students who have a lower level of test anxiety. |  | - Test-enhanced learning (formative assessment) |
| Preoteasa, Imre (53) | Romania | Dental students | The goal of the study is to compare dental students' psychological well-being during the summer semester examination period and the summer vacation. | - Prospective study (single arm) - 34 dental students participated. - The psychological well-being was assessed using the WHO-Five Wellbeing Index. | The semester examination period is likely to have a negative impact on psychological well-being on a large scale. | - Semester exam | - Summer holiday exam |
| Schwartz, Evans (54) | Canada | Physiotherapy students | The goal of the study was to see how much anxiety people felt during a timed and untimed test with similar content. | - Cross-sectional study - 81 students participated - State-Trait Anxiety Inventory (STAI) was used. | Students performed significantly better on the untimed test (P50.005), and test anxiety was significantly reduced (P0.001). Students who performed poorly on the timed test improved the most on the untimed test (x 520.4 610 percent ). The untimed test was preferred by 83 percent of students (n=69). | - Timed tests | - Untimed tests |
| Turan and Üner (55) | Turkey | Medical students | The goal of the study is to determine how much time interns spend preparing for the specialty exam, how the preparation process affects their training, and which factors are linked to test anxiety. | - Cross-sectional study - 214 intern students participated | The health status, economic level, perception of academic achievement, time allocated to study for the exam, time remaining until the exam, and trait anxiety level of participating interns all showed a correlation with exam anxiety. | - Health status - Economic level - Perception of academic achievement - Study time |  |
| Turner, Bartlett (56) | UK | Dental students | The goal of the study was to determine students' perceptions of study barriers and the relationship between these perceptions and demographic characteristics, perceived stress levels, and examination performance. | - Cross-sectional study - 90 students participated | Exam performance was significantly related to social distractions; students who rated social distractions highly performed worse. | - Social distraction |  |
| Bovee (57) | USA | Health professions students | The goal of the study was to look into the impact of collaborative testing on test anxiety in students at professional health institutions. | - Literature review. | Collaborative testing may assist in the reduction of test anxiety. |  | - Collaborative testing |
| Cobbett and Snelgrove-Clarke (58) | Canada | Nursing students | The researchers wanted to see how effective virtual clinical simulation and face-to-face high-fidelity manikin simulation were for maternal newborn clinical simulation. | - Experimental study (RCT) - 56 nursing students participated | Students in the virtual clinical simulation had higher anxiety levels than those in the face-to-face simulation. | - Virtual simulation | - Face-to face simulation |
| Crego, Carrillo-Diaz (59) | Spain | Dental students | The researchers wanted to see if there was a model that could explain the link between test anxiety and dental student performance. Students' strategies for dealing with stress during the exam period, as well as their self-efficacy assessments, were expected to influence the relationship between test anxiety and grades. | - Cross-sectional study - 201 students participated. - The researchers used an online survey that included questions about coping strategies, perceived stress, exam-related self-efficacy, and academic performance. | Emotional coping strategies (venting negative emotions, negative auto-focus) were linked to increased academic stress (=0.34, p0.01). Rational coping strategies (problem-solving, positive reappraisal, seeking social support) were negatively associated with perceived stress (=-0.25, p0.01). | - Emotional coping strategies (venting negative emotions, negative auto-focus) | - problem-solving, - positive reappraisal, - looking for social support) |
| Durning, Dong (60) | USA | Not determined | The goal of the study was to compare the relative utility of open-book and closed-book examinations (CBEs). | - Systematic review | Exam performance studies favored CBE, especially when CBE preparation was greater than OBE preparation. |  | - Closed book examinations |
| Green, Angoff (61) | USA | Medical students | The objectives of this study are to "(1) characterize test anxiety among students at one medical school; (2) determine the relationship between test anxiety and performance on the USMLE step-1 examination; and (3) determine the impact of a test-taking strategies course on test anxiety and USMLE scores." | - Experimental study (pre and post) - 93 students participated. - Westside Test Anxiety Scale was used. | The participants' test anxiety score decreased from 2.79 to 2.61 after the course (p = 0.09), and then to 2.53 after the USMLE (p = 0.02), whereas the controls' scores increased. |  | - Test taking strategy course |
| Kim (62) | South Korea | Medical students | The goal of the study was to look into the characteristics of medical students that were linked to their test anxiety on Objective Structured Clinical Examinations (OSCEs). | - Cross-sectional study - 94 students participated. | On the OSCE, participants' test anxiety had a moderate relationship with their class-related achievement emotions and a weak negative relationship with their patient-centeredness. | - Achievement emotions - Patient-centeredness OSCE |  |
| Klausenitz, Hacker (63) | Germany | Medical students | In a randomized crossover study, researchers wanted to see if Auricular Acupuncture (AA) could reduce exam anxiety in medical students when compared to placebo and no intervention. | - Experimental study (RCT) - A total of 44 medical students were randomly assigned to receive either AA, a placebo, or no treatment. - A visual analogue scale was used to assess anxiety levels. - The State-Trait-Anxiety Inventory was used as an additional criterion. | When compared to baseline and the no-intervention condition, anxiety levels were lower after the AA and placebo interventions (p 0.003). In the evening before the exam, AA was more effective than placebo at reducing anxiety. |  | - Auricular acupuncture |
| Tagher and Robinson (64) | USA | Nursing students | The purpose of this research is to look into the important aspects of how final-semester prelicensure nursing students perceive the stress of taking the Health Education Systems Incorporated Exit Examination (HESI E). | - Qualitative study (Focus group discussion) | There were six major categories of description that were discovered to increase test anxiety. | - Fear - Physical and psychological symptoms - Lack of balancing - Isolation - Motivation - Lack of consistency |  |
| Brodersen (65) | USA | Nursing students | The goal of the study was to find and evaluate TA interventions for nursing students. | - Review study (literature review). | Experimental evidence was found to support aromatherapy, music therapy, and a variety of cognitive and behavioral interventions. There was also nonexperimental evidence supporting collaborative testing, crib sheets, and amusing exam items. |  | - Collaborative testing - Crib sheets - Humorous exam items - Music therapy - Aromatherapy - Hypnotherapy - Rational stage-directed hypnotherapy - Relaxation training (progressive/deep muscle relaxation) - Systematic desensitization - Stress inoculation - Counseling for nutrition, exercise, relaxation, study skills, test-taking strategies - Biofeedback-assisted relaxation training - Guided reflection - Guided imagery training - Guided imagery with relaxation training - Aerobic exercise - Exam analysis - Pet therapy – certified therapy dog - Test-taking skills workshop |
| Fournier, Couret (66) | USA | Mix of health professions students | Through collaborative testing, the researchers wanted to learn more about the mechanisms that lead to improved performance. | - Cross-sectional study - 444 students participated - Before the first and last examinations, data on test anxiety was collected (using the Motivated Strategies for Learning Questionnaire). | Over the course of the semester, the majority of students reported a decrease in test anxiety. Test anxiety was higher in students with lower academic ability (often from minority ethnic groups) and women. Collaboration testing resulted in better grades. | - Female - Academic weak students | - Collaborative testing |
| Kalantari, Zadeh (67) | Iran | Dental students | The goal of the study was to see if the level of anxiety instilled in dental students at Kerman Dental School by the OSCE is higher than that induced by a written exam and a preclinical endodontic and crown preparation exam. | - Cross‐sectional study. - 138 students participated - Spielberger’s state anxiety inventory was used. | In all three assessment methods, state anxiety was high. Other assessment formats elicited less anxiety than the OSCE. | - OSCE |  |
| Khoshhal, Khairy (68) | Saudi Arabia | Medical students | The goal of the study was to determine the prevalence of exam anxiety among Taibah University medical students and to identify the factors that contributed to it. | - Cross-section study - 111 students participated - A visual analog scale was used to assess exam anxiety. | Exam anxiety affected approximately 65 percent of students for a variety of reasons. These were included in the list of factors that contribute to test anxiety. | - Female - Extensive course load - Long duration of exam - Inadequate rest - Irrational thoughts about exam and outcomes - No control over exam situation - Not studying - Memorize textbooks - Negative thinking and self-criticism - Do not recall and review - Studying all night before exam - Improper nutrition - Lack of physical exercise |  |
| Liu and Xu (69) | USA | Nursing students | The goal of the study was to learn more about American community college nursing students' experiences with using extended time and other forms of supportive accommodation to deal with test anxiety. | - Qualitative study (Focus group discussion). | Six distinct themes emerged. These are included in the list of factors that contribute to test anxiety. | - students’ feelings of being anxious and overwhelmed, - the impact of nursing school stress, - the perceived benefits of using test accommodations, - environmental influences, - challenges and supportive structures for securing accommodations, and - meta-perception of stigmatization |  |
| Massey, Byrne (70) | Australia | Nursing students | The goal of the study was to implement and evaluate an innovative approach to OSCE preparation in an undergraduate (registration) acute care nursing course. | - Mixed methods - 730 students participated | Video exemplars increased self-reported student confidence and understanding of performance expectations, resulting in increased engagement and decreased anxiety during OSCE preparation, but not overall OSCE performance. |  | - Video exemplar OSCE |
| Patil and Aithala (71) | India | Medical students | The goals are to (1) use the Westside test anxiety scale to determine the prevalence of exam anxiety among medical students, (2) evaluate any significant differences in exam anxiety levels between male and female students, and (3) assess the factors that cause exam anxiety. | - Cross-sectional study - 300 medical students participated. - Westside test anxiety scale was used. | The current study found that students in Phases I and III had higher rates of high exam anxiety. Under the heading "factors that increase test anxiety," there was a list of factors linked to test anxiety. | - Males - The examination system, - lack of time management, - extensive course load - Continuous examination with least gaps - Anxiety of oral examination - Studying all night before exam - Do not recall and review - Negative and irrational thinking about exams - Extensive course load - Inadequate rest - Inefficient studying - Frequent tests - More number of classes and home assignment - Lack strategic studying - Away from parents - Feelings of no control over exam situation - Parental pressure |  |
| Quinn and Peters (72) | USA | Nursing students | The goal of the study was to find interventions that would help prelicensure nursing students with test anxiety. | - Review study (literature review) | This systematic review of the literature identified interventions that can help prelicensure nursing students with test anxiety. |  | - Environmental Adjustments - Student Behavior Medication |
| Rice, Vogelweid (73) | USA | Veterinary students | The purpose of this study was to look at how veterinary students performed on exams using crib sheets to see how they affected exam performance, exam stress, and subject knowledge retention. | - Experimental study (pre and post) - Sample size was not mentioned clearly | Students viewed crib sheets as a way to reduce exam anxiety and provide support during studying and testing, according to the results of the survey. |  | - Crib sheet |
| Cai, Pan (74) | China | Medical students | The objective of this study was to see if attentional bias modification (ABM) can be used to change people with high test anxiety's attention to emotional information, and if this change is linked to anxiety vulnerability. | - Experimental study (RCT) - 77 students were participated. - As part of their ABM training, 28 people were given a 5-day modified dot probe task. - As a placebo, 29 people were given a 5-day classic dot probe task. - Between the two test sections, 20 people did not receive any intervention. | After the 5-day training, there was a significant change in attentional bias scores, whereas there were no changes in attentional bias scores in the placebo or waiting list groups. Importantly, the training group's anxiety vulnerability with regard to threats was significantly reduced. |  | - Attentional bias modification |
| Custer (75) | USA | Nursing students | The study's objectives are to: 1) ascertain the relationship between test anxiety and academic procrastination among prelicensure nursing students; 2) ascertain differences in test anxiety and academic procrastination across prelicensure nursing program types; 3) ascertain factors affecting the incidence of academic procrastination; and 4) ascertain the tasks most frequently procrastinated by prelicensure nursing students. | - Cross-sectional study - 202 students participated. - Test Anxiety Inventory (TAI), and the Procrastination Assessment Scale for Students (PASS) were used. | Correlations between test anxiety and academic procrastination were discovered that were statistically significant. | - Procrastination |  |
| Guraya, Guraya (76) | Saudi Arabia | Medical students | The purpose of this study is to objectively investigate and classify the perceptions of Saudi undergraduate medical students regarding test anxiety induced by various assessment tools. | - Cross-sectional study - 191 students participated. | The longest case exam resulted in the highest total anxiety score, followed by the examiner-based objective structured clinical examination. These were listed as factors that contribute to test anxiety. | - Long case - OSCE - Female - No time - No exercise - Irrational thoughts |  |
| Macauley, Plummer (77) | USA | Health professions students | The purpose of this study is to describe the prevalence of anxiety in healthcare professions students and to identify predictors of anxiety. | - Cross-sectional study - 183 students participated. - State-Trait Anxiety Inventory (STAI) and the Westside Test Anxiety Scale (WTAS) were used. | Females, financial support, poor academic performance, and pre-existing psychological conditions were all associated with increased test anxiety. | - Females - Financial support - Poor academic performance - Existing psychological problem |  |
| Kolagari, Modanloo (78) | Iran | Nursing students | The purpose of this study is to determine the effect of computer-based tests (CBT)on the test anxiety of nursing students. | - Quasi-experimental study - 39 nursing students participated. - Sarason’s Test Anxiety Scale (TAS) was used. | CBT has been shown to increase anxiety. | - CBT |  |
| Vanderoost, Janssen (79) | Belgium | Medical students | This is the first study to compare elimination testing with Arnold & Arnold's scoring rule (hereafter referred to as elimination testing with adapted scoring) and negative marking in detail. | - Mixed study design | Elimination testing with adapted scoring was preferred by students over negative marking, and students reported lower stress levels in elimination testing with adapted scoring than in negative marking. |  | - Adopted scoring |
| Zhang and Walton (80) | Canada |  | The goal is to gather information and conduct a descriptive content analysis of the primary causes of practical exam anxiety among Master's of Physical Therapy (MPT) students in a Canadian university program. | - Qualitative study (thematic analysis) - 56 students participated. | Six emergent meta-themes emerged from the thematic content analysis, which adequately captured all triggers. These were included in the list of factors that contribute to test anxiety. | - Social Performance Anxiety, - Fear of Lacking Competence, - Overvaluing the Outcome, - Fear of the Unknown, - Impaired Personal Health/Coping Resources, - Operational/Procedural Inﬂuences |  |
| Al-Sahman, Al-Sahman (81) | Saudi Arabia | Dental students | The goal of the study was to assess exam-related anxiety in undergraduate dental students and to determine the factors that contribute to exam anxiety. | - Cross-sectional study - 237 dental graduates participated | Excessive course load, fear of failure during the exam, and studying all night before exams were the main causes of exam anxiety. | - Excessive course load, - Fear of failure - Studying all night before exams. |  |
| Alammari and Bukhary (82) | Saudi Arabia | Dental students | The goal is to look into the prevalence of exam anxiety among prosthodontics students, as well as the factors that influence it. | - Cross-sectional study - 492 students participated - A self-administered questionnaire was used | The main factors influenced by gender were a lack of time to prepare for exams and an inability to recall information prior to exams. | - Lack of time - Inability to recall - Social media uses - Fear of failure |  |
| Burton and Baxter (83) | USA | Occupational therapy | The goal of this study is to see how coloring as a leisure activity affects students' perceptions of exam readiness and performance. | - Experimental study (pre and post) - 41 students participated - A self-report assessment was used. | Participants who engaged in the leisure activity of coloring showed a significantly lower level of anxiety than those who did not. |  | - Leisure activity (coloring) |
| Cipra and Müller-Hilke (84) | Germany | Health profession education students | The goal is to look into the development of exam-related anxiety and see if there is a link between anxiety and learning styles. | - Cross-sectional study - 212 medical students participated - State-trait-anxiety inventory (STAI-T) and the approaches-and-study-skills-inventory-for-students (ASSIST) were used. | The most important findings were that a surface learning approach was associated with anxiety as a trait, and that students who took a more strategic approach to learning were the least anxious while also being the most successful academically. | - Surface learner | - Strategic learner |
| Gilavand, Moezzi (85) | Iran | Dental students | The goal of the study was to look into test anxiety among dental students at Ahvaz Jundishapur University of Medical Sciences in Iran's southwest. | - Cross-sectional study - 160 students participated. - The standard questionnaire (TAI) (Test Anxiety Inventory) was used |  | - Gender, - Unsatisfaction with field of study - Age |  |
| Loya and Jiwane (86) | India | Medical student | The purpose of this study is to determine the level of exam anxiety experienced by professional medical students and the methods used to alleviate it prior to examination. | - Cross-sectional study - 387 students participated - A questionnaire was used. | The most common method used to relieve exam stress was contacting family and friends, which was used by 336(86.8%) students, followed by listening to music by 325(84.0%) students, and sleeping by 301(77.8%) students. |  | - Contacting family and friends - Listening music - Sleeping |
| Manansingh, Tatum (87) | USA | Nursing students | The goal is to see how relaxation techniques affect first-year baccalaureate nursing (BSN) students' academic stress, test anxiety, and desire to continue in the field. | - Mixed study design - A 6-week relaxation technique intervention and a focus group were conducted with 45 students. - A pretest was distributed the first week of class, followed by a focus group interview, and a posttest was distributed at the end of the semester. | According to the paired-sample t test, students who used relaxation techniques had lower academic stress and test anxiety. A thematic analysis of the focus group interview revealed that using relaxation techniques before exams helped to reduce mental and physical stress, as well as the fear of failure, and increased the likelihood that they would continue in the nursing profession. |  | - Relaxation techniques |
| Michael, Lyden (88) | USA | Sonographic students | The goal is to see how incorporating open book exams (OBEs) into sonography education affects students by using the testing method in two ultrasound physics courses. | - Mixed study design - A total of 17 students enrolled in a diagnostic medical sonography program made up the open-book subject population. | Overall, the findings of this study may support the use of OBEs in ultrasound physics courses in the future. |  | - Open-book exam |
| Poorman, Mastorovich (89) | USA | Nursing students | The goal is to present a number of practical interventions that faculty can use to assist students who are dealing with this debilitating issue. | - Review study (literature review) | This review identified a number of interventions. These were included in the list of factors that help with test anxiety. |  | - Cognitive - Restructuring, - Thought Stopping, - Earplugs, - Writing Questions, - Practicing Questions, - Progressive Relaxation" |
| Son, So (90) | South Korea | Nursing students | The goal is to determine the effects of aromatherapy and music therapy on test anxiety, state anxiety, stress, and basic nursing skills among Korean nursing students. | - Experimental study (RCT) - 98 students participated. | The findings suggest that aromatherapy combined with music therapy in nursing education may be effective in improving fundamental nursing skills and reducing anxiety and stress among nursing students. |  | - Aromatherapy - Music |
| Tsegay, Shumet (91) | Ethiopia | Medical students | The goal is to find out how common test anxiety is among medical students and what factors contribute to it. | - Cross-sectional study - 390 students participated. - Westside Test Anxiety Inventory (WTAI) was used. | This study discovered a link between test anxiety and female sex, a low GPA, being a first-year student, an excessive course load, an oral examination, a lack of a study plan, poor social support, moderate social support, and psychological distress. | - Female sex, - Having poor grade point average, - Being the first year, - Excessive course load, - Oral examination, - Lack of study plan, - Poor social support, - Moderate social support, - Having psychological distress |  |
| Warshawski, Bar-Lev (92) | Israel | Nursing students | The goal is to look into the relationships between test anxiety, academic self-efficacy (ASE), and social support from social media, as well as differences in test anxiety by year of study and cultural background. | - Cross-sectional study - 240 students participated. - A questionnaire was used to evaluate ASE | Lower test anxiety was linked to higher ASE and social media support. |  | - Academic self-efficacy - Social support |
| Yusefzadeh, Iranagh (93) | Iran |  | The purpose of this study is to see how study preparation affects test anxiety and performance in public health students. | - Quasi-experimental study - The intervention group consisted of (n=20) and the control group consisted of (n=25). | Test anxiety, both before and during exams, has a significant impact on students' grades. The intervention had a significant impact on test anxiety and exam scores. |  | - Refresher course - End session summary - Class presentation - Questions/answers |
| Zhang and Henderson (94) | USA | Chiropractic students | The goal of this study is to assess 1st-year chiropractic students' perceptions of stress and the relationship between stress and test anxiety. | - Cross-sectional study - 407 students participated. - The Center for Epidemiologic Studies Depression Scale and the Test Anxiety Inventory were used to assess pre-matriculation GPA, perceived chiropractic college stress (PCCS), the Center for Epidemiologic Studies Depression Scale, and the Test Anxiety Inventory. | Different factors were found to increase or decrease test anxiety. These were listed in the columns that corresponded to them. | - High academic expectations - Heavy workload, - Financial concerns. | - Financial counseling - Time management - Emphasizing content mastery - Incorporating active learning approaches |
| Akbari, Asayesh (95) | Iran | Paramedical students | The goal of this study is to see if having breakfast has anything to do with paramedical students' test anxiety. | - Cross-sectional study - 223 students were selected. - A self‑administered questionnaire was used. | Breakfast was skipped by 40.1 % of the time. Breakfast consumption was associated with lower test anxiety (OR: 0.54, 95 % CI: 0.30–0.95). | - Skipping breakfast |  |
| Naeim, Rezaeisharif (96) | Iran | Medical student | The goal is to see if there's a link between Internet addiction, social adjustment, and test anxiety among Ardabil University of Medical Sciences' female students. | - Cross-sectional study - 346 students participated. - Three questionnaires were used to collect data on Internet addiction, social adjustment, and test anxiety. | The findings revealed a link between Internet addiction and social adjustment, as well as test anxiety. | - Internet addiction - Social adjustment |  |
| REHMAN, SAEED (97) | Pakistan | Medical students | The purpose of this study is to look into the factors that influence Exam Anxiety among Medical Students in Lahore. | Cross-sectional study | The results revealed that there was no difference in these factors between male and female students. Males reported doing more physical activity during the exam (p=0.001). During exams, slightly more females than males used antidepressants (p=0.003). They had more negative thoughts and self-criticism (p=0.005) and paid less attention to their diet during exams (p=0.004) than males. | - Females - Use anti-depressants - Negative thinking - Self-criticism during exam - Nutrition - Strained relations - Away from home - Finances | - Using exercise |

For each class, the related factors were further categorized into themes according to their relevance (Table S5 and S6).

Table S5: Themes of factors that increase test anxiety

| Themes | Category | Sub-categories (if there) | References (cited studies) |
| --- | --- | --- | --- |
| Assessment system | Format | OSCE | (28, 36, 44, 62, 67, 76, 77, 80) |
|  |  | Oral | (71, 91) |
|  |  | Computer-based | (78) |
|  |  | Long case | (76) |
|  |  | Virtual simulation | (58) |
|  | Logistics | Problems in test construction | (64, 68, 71) |
|  |  | Clarity of task being assessed | (62, 80) |
|  |  | Long duration of test | (33, 68) |
|  | Policy/regulations of assessment | Frequent test with short gaps | (53, 54, 71) |
|  |  | Fixed dates of tests | (53, 54, 71) |
|  |  | High stakes | (46, 55) |
| Study and learning skills | Lack of learning skills | Inability to recall | (68, 71, 82) |
|  |  | Surface learner | (84) |
|  | Lack of study skills | Inefficient studying | (71) |
|  |  | Lack of exam taking tips | (33) |
|  |  | Lack of study plan | (91) |
|  |  | Memorize textbooks | (68) |
|  | Improper time management | Procrastination | (64, 71, 75, 76, 82) |
|  |  | Study all night before exam | (68, 71, 81) |
| Psychology/cognition | Irrational thoughts and believes | Negative thoughts | (41, 59, 64, 68, 69, 71, 76, 80-82, 97) |
|  | Previous illness | Existing psychological distress | (64, 69, 77) |
|  | Self-expectation | High expectation | (62, 94) |
| Personal characteristics | Gender | Female | (33, 34, 43, 66, 68, 76, 77, 85, 91, 97) |
|  |  | Male | (71) |
|  | Past academic performance | History of poor academic performance | (24, 66, 77, 91) |
|  | Age | Young age | (24, 85, 91) |
|  | Self-care | Neglecting breakfast/ nutrition | (68, 95, 97) |
|  |  | Lack of physical activity | (68, 76) |
|  | Addiction | Social media and internet addition | (82, 96) |
|  |  | Drug addiction | (97) |
|  | Personality type | Extraversion and neuroticism | (34) |
| Support and relationship | Loss of social support | Strained relationship and social distraction | (56, 91, 97) |
|  | Lacking financial support |  | (77, 94, 97) |
|  | Lacking parental support | Away from home | (71, 97) |
|  |  | Parent high expectation | (71) |
| Curriculum | Course load | Excessive course load | (31, 33, 71, 81, 91, 94) |
|  | Assignment | More number of classes and home assignment | (71) |
|  | School environment | Accommodation issues | (69) |

Table S6: Themes of factors that decrease test anxiety

| Themes | Categories | Sub-categories (if there) | References |
| --- | --- | --- | --- |
| Assessment system | Assessment approach | Open book test | (26, 43, 60, 88) |
|  |  | Collaborative testing | (57, 65, 66) |
|  |  | Face to face simulation | (58) |
|  |  | Peer-assisted mock OSCE | (48) |
|  |  | Progress testing | (50) |
|  |  | Rationales of Q&A | (27) |
|  |  | Test-enhanced learning | (52) |
|  |  | Video exemplar OSCE | (70) |
|  |  | Video-based learning activity | (40) |
|  |  | Participating in writing questions | (89) |
|  | Logistics | Crib sheet | (65, 73) |
|  |  | Suitable test time for students | (53, 54) |
|  |  | Injecting humor items | (65) |
|  | Scoring and grading methods | Pass-fail grading | (30, 35, 38, 43, 49) |
|  |  | Elimination testing with adopted scoring (no negative marks) | (79) |
|  |  | Exam analysis (discussion on test items after exam) | (65) |
| Study and learning skills | Improving learning skills | Effective leaning strategies | (29, 61, 65, 84, 94) |
|  |  | Content mastery | (94) |
|  |  | Active learning | (94) |
|  | Improving study skills | Test taking strategies | (47, 65) |
|  |  | Practicing questions | (89, 93) |
|  |  | Comprehensive reading | (27) |
|  |  | Note taking | (27) |
|  |  | Class presentation | (93) |
|  |  | Group study | (27) |
|  | Time management | Improving time management skills | (27, 29, 31, 94) |
| Psychology/cognition | Behavioral modifications | Behavior modification | (24, 39, 45, 59, 72, 74) |
|  |  | Desensitization | (45, 65) (47) |
|  | Cognitive modifications | Cognitive re-structuring | (41, 51, 65, 89, 92) |
|  |  | Guided imagery | (27, 65) |
| Personal characteristics | Self-care | Sport | (41, 65) |
|  |  | Nutrition | (65) |
| Support and relationship | Institutional | Providing a short review course | (32, 93, 94) |
|  |  | Academic counselling | (45) |
|  |  | Financial support | (94) |
|  | Family/friends | Emotional/advising support | (24, 86, 92) |
| Interventional procedures | Meditation intervention | Relaxation techniques including deep breathing and yoga | (25, 27, 47, 65, 87, 89) |
|  | Sensory interventions (smelling, hearing and visual) | Music | (47, 65, 86, 90) |
|  |  | Aromatherapy (lemon oil) | (42, 47, 65, 90) |
|  |  | Earplug | (89) |
|  |  | Coloring leisure activity | (83) |
|  | Other interventions | Hypnotherapy | (47, 65) |
|  |  | Mind sound technology | (37) |
|  |  | Pet | (65) |
|  |  | Auricular acupuncture | (63) |

# **Appendix III: (Focus Group Discussion)**

Aim:

The purpose of the focus group discussion (FGD) was to elicit students' knowledge, perspectives, and attitudes concerning test anxiety (TA) and their strategies for dealing with it.

####

#### Sample size:

In FGD, the sample size was estimated using the saturation principle (98, 99). Saturation was defined as the absence of new information elicited in the additional FGD and the repetition of similar input (98).

#### Participants, sampling, and recruitment

Students from different academic years were approached for participation via invitations sent by their academic year's group leaders. WhatsApp messages were also used containing information about the study and a link of consent form. A token was assigned to each participant after their session. Purposive sampling was used to obtain a diverse range of experiences, considering student diversity such as gender and race (e.g., Malay, Chinese, Indian, or other).

#### Data collection

We piloted the FGD protocol with a group of students, and they reported that it was open-ended and stimulated discussion. Next, the FGD sessions were conducted in a quiet and comfortable room. Each session was beginning by welcoming the group members and briefing them on the study’s purpose. To provide a comfortable environment, each session was conducted in a private meeting room. A circular seating arrangement was set to promote sense of equality among the participants. Each participant’s pseudonym (nickname) was tagged in front of each seat to ensure participants anonymity and to facilitate group interaction. Based on the predetermined probe questions, the researcher initiated the audiotaped discussion with an open-ended cue: “Test anxiety to me is…”. Notes were made to reflect non-verbal cues. Each FGD ended after 60 to 90 minutes. Data collection continued until saturation was reached.

#### Data analysis

The data analysis process began concurrently with the data collection process. This interim analysis aided the researcher in fine-tuning and validating the emerging themes considering the subsequent data. The method of Braun and Clark's six-phase thematic analysis was used (100).

#### Emerged themes and subthemes:


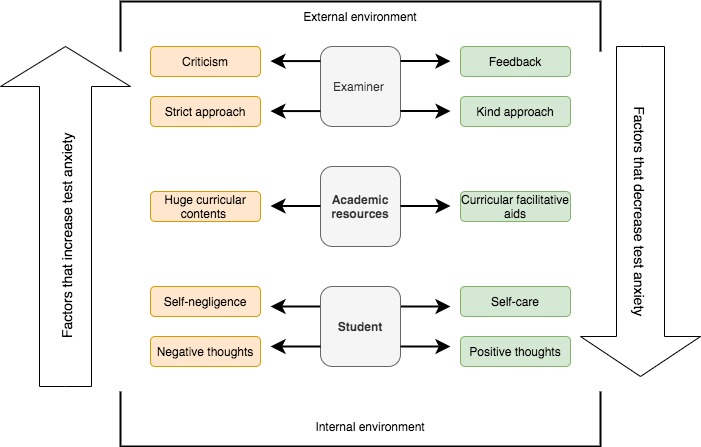
Students, academic resources, and examiners were the three major themes that emerged from the analysis of FGD (Figure S2). Each theme was subdivided into subthemes that showed a rise or fall in TA.

Figure S2: Emerged themes and sub-themes in relation to increasing and decreasing test anxiety

The gray-colored themes were positioned in the center of the figure. All subthemes with a rise in TA were grouped and colored red, whereas all subthemes with a decrease in TA were grouped and colored green. The arrows on either side of the picture represent the two primary probe questions posed throughout the FGD. Notably, the themes and their associated subthemes were arranged from exterior to interior to represent their interrelationships.

The corresponding quotations for each sub-theme are shown in Table S7.

Table S7: Emerged themes and sub-themes from FGD with supporting quotations

| **Themes** | **Increasing TA** | | **Decreasing TA** | |
| --- | --- | --- | --- | --- |
|  | **Sub-themes** | **Quotations** | **Sub-themes** | **Quotations** |
| Student | Negative thoughts | “The negative talk about exam by classmates increase anxiety” (Student 5, group C) | Positive thoughts | “I do believe that professional exam is the worst feeling ever in life, this worst feeling reduced by always practice positive thoughts, this worst feeling reduced by early planning” (Student 2, group A) |
|  | Self-negligence | “I changed my whole sleep cycle. I went to sleep at 7:00 am and wake up at 1:00 pm. I drink two cups of coffee per day. I knew some of my friend drink 5 – 10 cups of coffee. These kinds of things are not normal to your body and increase my stress”. (Student 4, group B) | Self-care | “I need adequate sleep. Especially night sleep even one day before exams. I can’t stay up at night although if I’m nervous  And secondly, what really helps me with stress or anxiety is good food and sleep”. (Student 2, group C) |
| Academic resources | Huge curricular contents | “We already have a huge syllabus to go through”. (Student 1, group B)  “I feel stupid as I did not cover contents. It makes me nervous”. (Student 6, group F) | Curricular facilitative aids | “I would suggest that, besides having all these major examination like OSCE or theory paper, we can include continue assessment in between so the lecturers can access what we have learned and at the same time we will have the motivation to study consistently and to avoid more stressful situations during major examination”. (Student 3, group B)  “Give quiz/homework based on learning outline in the end of every lecture so the students know what exactly they have to cover for each subject”. (Student 1, group A) |
| Examiner | Strict approach | “I add experience, I got to know that malignant doctor will examine me next day. Eight of students failed. The day I know is stressful, after that I switched to another type of thinking in which to take this challenge. So how he will tackle me and how prevent myself to tackle out”. (Student 4, group E) | Kind approach | “Actually, for both exams I got the same marks. But, I was feeling better during the first exam where the lecture calm me and after the exam she guided me about the question, even though I cannot answer it, she guided me what to do after that rather than to scold me and ask me to go out”. (Student 2, group F) |
|  | Criticism | “I just fear of being scolded during the exam”. (Student 6, group B)  “In the exam, we are just to assess student, we should not scold student”. (Student 5, group B) | Feedback | “Analyze each students' performances and try to discuss with student who has serious problem w that subject personally”. (Student 3, group A)  “I will teach him how to do correctly at that time then I will say the student will … happier and after exam he will tell other friends that examiner teach him”. (Student 2, group D) |

# **Appendix IV: (Generating resilience guidelines based on the scoping review and FGD )**

**
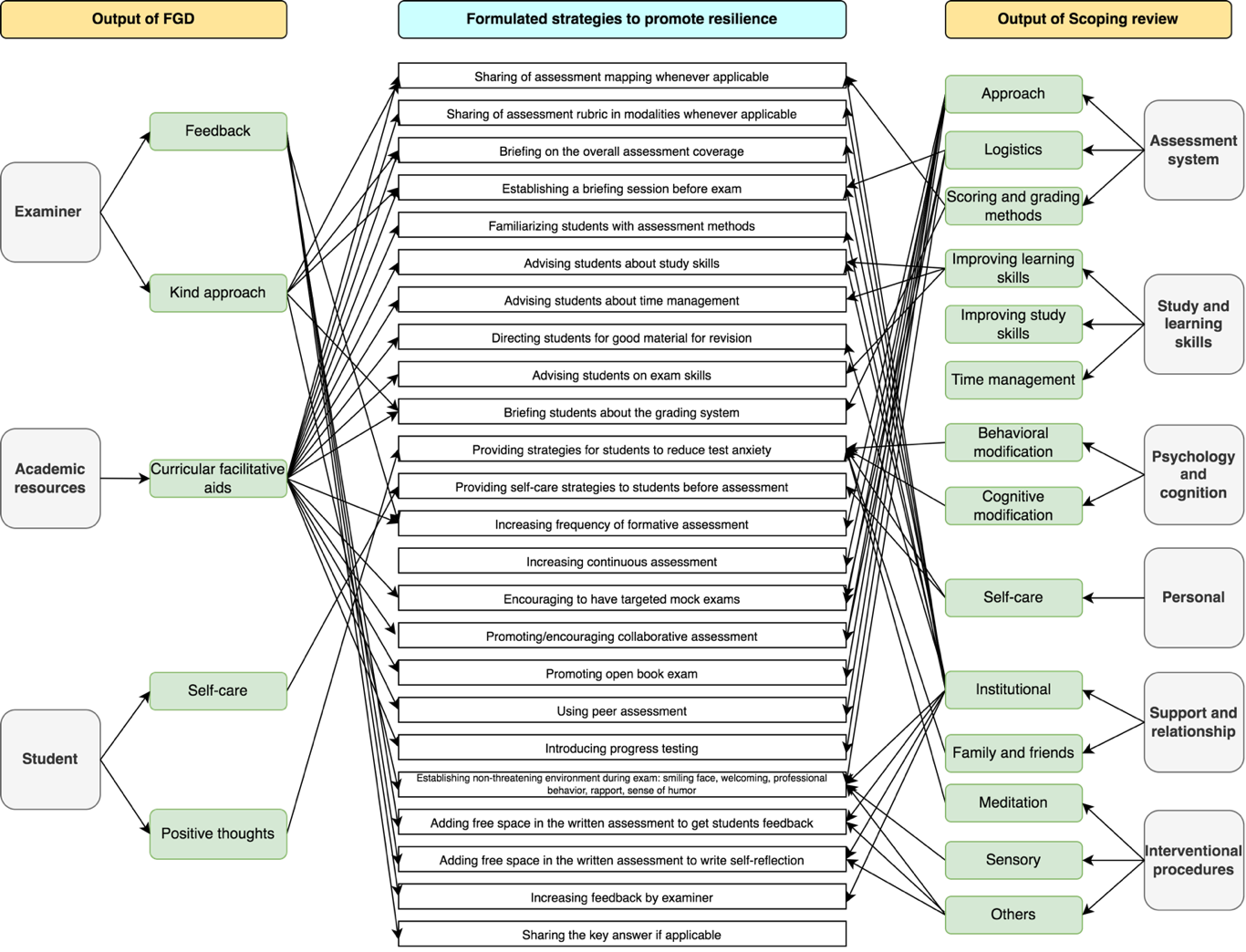
**

Figure S3: Relation of the proposed guidelines with outputs of scoping review and FGD

# **Appendix V: (Content Validation)**

**General Information:**

Content validation refers to the extent to which each SAR strategy adequately represents the point of interest during content distribution (101, 102). The researcher validated the content using the Content Validity Index (CVI). This method reflects the proportional agreement in which two or more expert panels independently assess the relevance of contents of the model to the domain of interest using a structured procedure (101).

Six to ten expert panels are recommended in performing CVI (101). The researcher invited ten expert panels comprised of individuals with substantial experience in Medical Education and student assessment.

The researcher created an inventory to assess the relevancy of the SAR strategies in relation to the SAR domains. According to Grant and Davis (103), the researcher used a four-point Likert scale to assess relevancy of each strategy to its corresponding area. For clarification selecting 1 means the item is irrelevant, while 4 means the item is extremely relevant. All domains and significant terminologies were defined, and each item in the inventory was elaborated.

Three types of CVI that were calculated; item/strategy-level CVIs (I-CVIs), scale/model-level CVIs (S-CVI) using the universal agreement method (S-CVI/UA), and average calculation method (S-CVI/Ave) (101, 104, 105). In I-CVIs, the experts rated the relevance of each strategy of the model to the resilience domains. Using a dichotomous rating of relevance, the experts ratings of 1 or 2, which indicating non-relevant, were considered as 0, while ratings of 3 and 4, which indicating relevant, were considered as 1 (104). In S-CVI/UA, those SAR strategies receiving a rating of 3 or 4 (relevant) by all expert panels were calculated by its proportion. In S-CVI/Ave, the average score of all I-CVIs for all strategies was calculated (104). Table S8 illustrates the template of CVI rating utilized in this study and Table S9 summarizes the acceptable value of I-CVIs, S-CVI/UA and S-CVI/Ave.

Table S8: Content Validation Index - Guidelines rated as 3 or 4 (relevant) is ticked on the table

| SAR strategies | Expert 1 | Expert 2 | Expert n | Number of experts in agreement | I-CVIs (Number of experts in agreement / Total number of experts) |
| --- | --- | --- | --- | --- | --- |
| Guideline 1 |  |  |  |  |  |
| Guideline 2 |  |  |  |  |  |
| Guideline n |  |  |  |  |  |
|  |  |  |  | S-CVI/UA (proportion of strategies with 'all- relevant' rating) = | S-CVI/Ave (average of I-CVIs) = |

Table S9: Acceptable values for content validity indices (104)

| **Indices** | **Number of panels** | **Acceptable values** |
| --- | --- | --- |
| I-CVIs | Five or fewer panels | 1.00 |
|  | Six to eight panels | 0.83 and above |
|  | Nine or more panels | 0.78 and above |
| S-CVI/UA |  | 0.80 and above |
| S-CVI(Ave) |  | 0.80 and above |

**Results:**

Table S10 summarized the demographic data of the expert panels. Six expert panels responded to the invitation. Majority of expert panels are males (67%), medical educationists (67%), and have extensive experience in medical education (67%). Other of experts are from educational sciences (33%).

The demographics of the expert panels were summarized in a table. Six expert panels responded to the invitation with response rate 60%. The majority of expert panels consist of males (67%), medical educators (67%), and individuals with extensive experience in medical education (67%). Other experts hail from the educational sciences (33%).

Table S10 Demographic data of the expert panels.

| **Demographic characteristics (n = 6)** | | **N (%)** |
| --- | --- | --- |
| **Gender** | |  |
|  | Male | 4 (67%) |
|  | Female | 2 (33%) |
| **Background** | |  |
|  | Medical Education | 4 (67%) |
|  | Educational Sciences | 2 (33%) |
| **Years of experiences** | |  |
|  | 5 – 10 years | 2 (33%) |
|  | >10 years | 4 (67%) |

Based on the values of CVI (Table S‎4‑13), 19 out of 24 guidelines (79%) achieved I-CVI of 1.00 (Table S‎4‑14). Based on the feedback of the experts and evaluating those guidelines with I-CVI values less than 0.80, five items (highlighted in Table S11) were removed before the framework went through a face validation study.

Table S11: The original version of the SAR guidelines that was sent for content validation

| SAR strategies (n=24) | Expert 1 | Expert 2 | Expert 3 | Expert 4 | Expert 5 | Expert 6 | Experts in agreement |  | I-CVI | UA |
| --- | --- | --- | --- | --- | --- | --- | --- | --- | --- | --- |
| 1. Sharing of assessment mapping whenever applicable | 1 | 1 | 1 | 1 | 1 | 1 | 6 |  | 1.0 | 1 |
| 1. Sharing of assessment rubric in modalities whenever applicable | 1 | 1 | 1 | 1 | 1 | 1 | 6 |  | 1.0 | 1 |
| 1. Briefing on the overall assessment coverage | 1 | 1 | 1 | 0 | 1 | 1 | 5 |  | 0.8 | 1 |
| 1. Establishing a briefing session before exam | 1 | 1 | 1 | 1 | 0 | 1 | 5 |  | 0.8 | 1 |
| 1. Familiarizing students with assessment methods | 1 | 1 | 1 | 1 | 1 | 1 | 6 |  | 1.0 | 1 |
| 1. Increasing frequency of formative assessment | 1 | 1 | 1 | 1 | 1 | 1 | 6 |  | 1.0 | 1 |
| 1. Increasing continuous assessment | 1 | 1 | 1 | 1 | 0 | 0 | 4 |  | 0.7 | 0 |
| 1. Encourage to have targeted mock exams | 1 | 1 | 1 | 1 | 1 | 0 | 5 |  | 0.8 | 1 |
| 1. Promoting/encouraging collaborative assessment | 1 | 1 | 1 | 1 | 1 | 1 | 6 |  | 1.0 | 1 |
| 1. Promoting open book exam | 1 | 1 | 1 | 1 | 1 | 1 | 6 |  | 1.0 | 1 |
| 1. Using peer assessment | 1 | 1 | 1 | 1 | 0 | 1 | 5 |  | 0.8 | 1 |
| 1. Introducing progress testing | 1 | 1 | 1 | 1 | 0 | 1 | 5 |  | 0.8 | 1 |
| 1. Advising students about study skills | 1 | 1 | 1 | 1 | 0 | 0 | 4 |  | 0.7 | 0 |
| 1. Advising students about time management | 1 | 1 | 1 | 1 | 0 | 1 | 5 |  | 0.8 | 1 |
| 1. Directing students for good material for revision | 1 | 1 | 1 | 1 | 1 | 1 | 6 |  | 1.0 | 1 |
| 1. Advising students on exam skills | 1 | 1 | 1 | 1 | 0 | 1 | 5 |  | 0.8 | 1 |
| 1. Briefing students about the grading system | 1 | 1 | 1 | 0 | 0 | 1 | 4 |  | 0.7 | 0 |
| 1. Providing strategies for students to reduce test anxiety | 1 | 1 | 1 | 1 | 1 | 1 | 6 |  | 1.0 | 1 |
| 1. Providing self-care strategies to students before assessment | 1 | 1 | 1 | 0 | 0 | 1 | 4 |  | 0.7 | 0 |
| 1. Establishing non-threatening environment during exam: smiling face, welcoming, professional behavior, rapport, sense of humor | 1 | 1 | 1 | 0 | 1 | 1 | 5 |  | 0.8 | 1 |
| 1. Adding free space in the written assessment to get students feedback | 1 | 1 | 1 | 0 | 0 | 0 | 3 |  | 0.5 | 0 |
| 1. Adding free space in the written assessment to write self-reflection | 1 | 1 | 1 | 1 | 0 | 1 | 5 |  | 0.8 | 1 |
| 1. Increasing feedback by examiner | 1 | 1 | 1 | 1 | 0 | 1 | 5 |  | 0.8 | 1 |
| 1. Sharing the key answer if applicable | 1 | 1 | 1 | 1 | 1 | 1 | 6 |  | 1.0 | 1 |
| Total of items | 24 | 24 | 24 | 19 | 12 | 21 |  | **S-CVI/Ave** | **0.85** |  |
| Proportional relevance | 1 | 1 | 1 | 0.79 | 0.5 | 0.875 |  | **S-CVI/UA** |  | **0.83** |
| **S-CVI/Ave** | **0.85** | | | | | |  |  |  |  |

# **Appendix VI: (Response Process)**

**General Information:**

The response process validity is the process by which the model and its strategies are evaluated in terms of their appropriateness, sensibility, and relevance from the perspective of users (106). Due to several concerns, including the fallibility of judgments based on superficial aspects of the content, experts have discouraged the use of the term 'face validity,' which was previously known as 'face validity' (107). Despite its detractors, the Face Validation Index (FVI), which employs a similar approach to the CVI, could be used to evaluate the validity of response processes (106).

The response process evaluation was performed via a group of medical teachers to measure their opinions on the clarity of language used for each strategy, and feasibility of SAR model. The group should not be less than 10 participants (106). For this reason, 20 participants were invited and approached via email. The email contains clear description about how the validation will be run. A video material was prepared describing how SAR could be applied into assessment practice and sent with invitation email. A response process form was also attached to the email. The medical teachers were asked to review all strategies and rate them based on its clarity and comprehensibility against a four-point scale (1 - not clear and comprehensible, 2 - somewhat clear and comprehensible, 3 - clear and comprehensible, 4 - very clear and comprehensible).

The researcher calculated three FVI indices: item/strategy FVIs (I-FVIs), scale/model FVIs using the universal agreement method (S-FVI/UA), and scale/model FVIs using the average method (S-FVI/UA) (Ave). To begin, all ratings were converted to a dichotomous scale: not-clear (ratings of 1 and 2) and clear (ratings of 3 and 4). The researcher then calculated the percentage of medical teachers who gave each strategy a 'clear' rating (I-FVIs). The proportion of strategies receiving a rating of 3 or 4 (clear) from all medical teachers was then determined in S-FVI/UA. The average score of all I-FVIs for all strategies was calculated in S-FVI/Ave. According to a review, the lower limit of an acceptable FVI is between 0.80 and 0.83 (106), and the researcher chose 0.83 as the lower limit of an acceptable FVI based on ratings from 10 health care workers (108). The template for the FVI rating used in this study is shown in Table S12.

Table S12: Content Validation Index - Guidelines rated as 3 or 4 (relevant) is ticked on the Table S

| SAR strategies | Rater 1 | Rater 2 | Rater n | Number of medical teachers in agreement | I-FVIs (Number of medical teachers in agreement / Total number of the group) |
| --- | --- | --- | --- | --- | --- |
| Strategy 1 |  |  |  |  |  |
| Strategy 2 |  |  |  |  |  |
| Strategy n |  |  |  |  |  |
|  |  |  |  | S-FVI/UA (proportion of topics with 'all- clear' rating) = | S-FVI/Ave (average of I-FVIs) = |

In a similar vein to CVI, the researcher included an open-ended column for medical educators to provide feedback on each strategy and the SAR model in general. The researcher then refined the SAR model further by incorporating the FVI ratings and open-ended feedback.

**Results:**

Table S13 provided a summary of the demographic information of the face validation panels. Twelve panels responded to the invitation (the response rate was 60%). The panels consisted of medical educators from various disciplines and universities. 42% were clinical educators, and 67% have more than ten years of teaching experience.

Table S13: Demographic data of the panels in face validation study.

| **Demographic characteristics (n = 12)** | | **N (%)** |
| --- | --- | --- |
| **Gender** | |  |
|  | Male | 5 (42%) |
|  | Female | 7 (58%) |
| **Background** | |  |
|  | Basic Biomedical Sciences | 7 (58%) |
|  | Clinical Sciences | 5 (42%) |
| **Years of experiences** | |  |
|  | 5 – 10 years | 4 (33%) |
|  | >10 years | 8 (67%) |

Based on the values of FVI Table S14, 17 out 19 items (89%) achieved I-FVI of 1.00. Based on the feedback of the medical teachers and evaluating those guidelines with I-CVI values less than 0.80, two items (highlighted in Table S14) were removed before the framework went through the real implementation.

| SAR strategies (n=19) | **Med. teacher 1** | **Med. teacher 2** | **Med. teacher 3** | **Med. teacher 4** | **Med. teacher 5** | **Med. teacher 6** | **Med. teacher 7** | **Med. teacher 8** | **Med. teacher 9** | **Med. teacher 10** | **Med. teacher 11** | **Med. teacher 12** | **Med. teachers** in agreement |  | **I-FVI** | UA |
| --- | --- | --- | --- | --- | --- | --- | --- | --- | --- | --- | --- | --- | --- | --- | --- | --- |
| 1. Sharing of assessment mapping/blueprinting whenever applicable | 1 | 1 | 1 | 1 | 1 | 1 | 1 | 1 | 1 | 1 | 1 | 1 | **12** |  | **1.00** | 1 |
| 1. Sharing of assessment rubric in modalities whenever applicable | 1 | 1 | 1 | 1 | 1 | 0 | 1 | 1 | 1 | 1 | 1 | 1 | **11** |  | **0.92** | 1 |
| 1. Briefing on the overall assessment coverage | 1 | 1 | 1 | 1 | 1 | 1 | 1 | 1 | 1 | 1 | 1 | 1 | **12** |  | **1.00** | 1 |
| 1. Establishing a briefing session before exam | 1 | 1 | 1 | 1 | 1 | 1 | 1 | 1 | 1 | 1 | 1 | 1 | **12** |  | **1.00** | 1 |
| 1. Familiarizing students with assessment methods | 1 | 1 | 1 | 1 | 1 | 1 | 1 | 1 | 1 | 1 | 1 | 1 | **12** |  | **1.00** | 1 |
| 1. Advising students about time management and study skills | 1 | 1 | 1 | 1 | 1 | 1 | 1 | 1 | 1 | 1 | 1 | 1 | **12** |  | **1.00** | 1 |
| 1. Directing students for good material for revision | 1 | 1 | 1 | 1 | 1 | 0 | 1 | 1 | 1 | 1 | 1 | 1 | **11** |  | **0.92** | 1 |
| 1. Advising students on exam skills | 1 | 1 | 1 | 1 | 1 | 1 | 1 | 1 | 1 | 1 | 1 | 1 | **12** |  | **1.00** | 1 |
| 1. Providing strategies for students to reduce test anxiety | 0 | 1 | 1 | 1 | 0 | 1 | 1 | 1 | 1 | 1 | 1 | 1 | **10** |  | **0.83** | 1 |
| 1. Increasing frequency of formative assessment | 1 | 1 | 1 | 1 | 1 | 1 | 1 | 1 | 1 | 1 | 1 | 1 | **12** |  | **1.00** | 1 |
| 1. Encourage to have targeted mock exams | 1 | 1 | 1 | 1 | 1 | 1 | 1 | 1 | 1 | 1 | 1 | 1 | **12** |  | **1.00** | 1 |
| 1. Promoting/encouraging collaborative assessment | 1 | 1 | 1 | 1 | 0 | 1 | 0 | 1 | 1 | 1 | 1 | 1 | **10** |  | **0.83** | 1 |
| 1. Promoting open book exam | 1 | 1 | 1 | 1 | 0 | 1 | 0 | 1 | 1 | 1 | 1 | 1 | **10** |  | **0.83** | 1 |
| 1. Using peer assessment | 1 | 1 | 1 | 1 | 0 | 0 | 1 | 1 | 1 | 1 | 1 | 1 | **10** |  | **0.83** | 1 |
| 1. Introducing progress testing | 1 | 1 | 1 | 1 | 1 | 0 | 0 | 0 | 1 | 1 | 1 | 1 | **9** |  | **0.75** | 0 |
| 1. Establishing non-threatening environment during exam | 1 | 1 | 1 | 1 | 1 | 1 | 1 | 1 | 1 | 1 | 1 | 1 | **12** |  | **1.00** | 1 |
| 1. Increasing feedback to examinee | 1 | 1 | 1 | 1 | 1 | 1 | 1 | 1 | 1 | 1 | 1 | 1 | **12** |  | **1.00** | 1 |
| 1. Sharing the key answer if applicable | 0 | 1 | 1 | 0 | 0 | 1 | 1 | 1 | 1 | 1 | 1 | 1 | **9** |  | **0.75** | 0 |
| 1. Adding free space/window for self-reflection | 1 | 1 | 1 | 1 | 0 | 1 | 0 | 1 | 1 | 1 | 1 | 1 | **10** |  | **0.83** | 1 |
| Total of items | **17** | **19** | **19** | **18** | **13** | **15** | **15** | **18** | **19** | **19** | **19** | **19** |  | **S-FVI/Ave** | **0.92** |  |
| Proportional relevance | **0.89** | **1.00** | **1.00** | **0.95** | **0.68** | **0.79** | **0.79** | **0.95** | **1.00** | **1.00** | **1.00** | **1.00** |  | **S-FVI/UA** |  | **0.89** |
| **S-FVI/Ave** | **0.92** | | | | | | | | | | | |  |  |  |  |

Table S14: The FVI indices of the 19 SAR guidelines

# **Appendix VII: (Written response of medical teacher in using SAR guideline)**

Table S15: The medical teachers’ feedback after response process

| Teachers | Quotations |
| --- | --- |
| Teacher 1 | “The provisional strategies for promoting resilience are clear and applicable. Meanwhile, I believe that promoting resilience relies on transparency and clarity.  I suggest increasing the role of students in establishment the assessment strategies through surveys or involvement of some students in the meeting about assessment policy with staffs”. |
|  |  |
| Teacher 4 | “I think it is an outstanding advancement in student assessment and it introduces excellent support to students”. |
|  |  |
| Teacher 5 | “Thank you for giving me the opportunity to provide feedback on this innovative model . This work is really excellent, and I appreciate all the efforts you have put into developing this model.  I just want to add a few explanatory notes to my responses on this model; Where my responses indicated 4: I meant the strategy was clear in the sense that it is relevant and clear how it can be applied and evaluated to improve student resilience. on the other hand, responses with score of 2s; I felt that it was not quite clear how are those strategies/items going to be implemented, measured and evaluated in a practical way.  One recommendation I would make to enhance clarity on those items is to revise the action verbs used to describe them. For instance, instead of saying "Establishing non-threatening environment during exam" which is a broad statement, I would suggest " Training examiners on how to make exams less anxiety-provoking”. So, the key here is to invest in examiners` training in a standardized way rather than to make a broad, non-specific statement. Once again thanks for the opportunity and good luck”. |
|  |  |
| Teacher 8 | “The above model is a very good model of resilience. However, while it may apply to some courses, it may not to others. It is important to distinguish its linkage to either theory, practical or clinical courses to make it more context specific. Good luck and do share your final results!” |
|  |  |
| Teacher 9 | “SAR is a unique, clear and motivating model. It inspires students to perform better and supports assessment for learning. Moreover, it helps students to learn more about themselves, their weakness and areas that needs improvement. I think, it will not improve academic achievement only, but it will make students life easier”. |
|  |  |
| Teacher 10 | “Thank you for giving me this opportunity to look at you great work. It was clear, comprehensive and focused. i as able to imagine it applied in by course. The supporting videos were informative. I think adding more clarity on what you mean by formative Vs mock exam would be helpful”. |
|  |  |
| Teacher 12 | “Very comprehensive excellent model” |

**References:**

1. Martin A. Motivation and Academic Resilience: Developing a Model for Student Enhancement. Australian Journal of Education. 2002;46(1):34-49. <http://doi.org/10.1177/0739986391013100210.1177/000494410204600104>

2. Martin AJ, Marsh HW. Academic resilience and its psychological and educational correlates: A construct validity approach. Psychology in the Schools. 2006;43(3):267-81. <https://doi.org/10.1002/pits.20149>

3. Dunn LB, Iglewicz A, Moutier C. A conceptual model of medical student well-being: promoting resilience and preventing burnout. Academic Psychiatry. 2008;32(1):44-53. <https://doi.org/10.1176/appi.ap.32.1.44>

4. Martin AJ, Marsh HW. Academic buoyancy: Towards an understanding of students' everyday academic resilience. Journal of School Psychology. 2008;46(1):53-83. <https://doi.org/10.1016/j.jsp.2007.01.002>

5. Kunicki ZJ, Harlow LL. Towards a Higher-Order Model of Resilience. Social Indicators Research. 2020;151(1):329-44. <https://doi.org/10.1007/s11205-020-02368-x>

6. Van Der Vleuten CPM. The assessment of professional competence: Developments, research and practical implications. Advances in Health Sciences Education. 1996;1(1):41-67. <https://doi.org/10.1007/BF00596229>

7. Gibbs G, Simpson C, Macdonald R, editors. Improving student learning through changing assessment–a conceptual and practical framework. European Association for Research into Learning and Instruction Conference, Padova, Italy; 2003: Citeseer. (Retrieved from: <https://citeseerx.ist.psu.edu/document?repid=rep1&type=pdf&doi=ca609b98befc83caf868ca6c28226cc8acc44d51>).

8. Baartman LK, Bastiaens TJ, Kirschner PA, Van der Vleuten CP. The wheel of competency assessment: Presenting quality criteria for competency assessment programs. Studies in Educational Evaluation. 2006;32(2):153-70. <https://doi.org/10.1016/j.stueduc.2006.04.006>

9. Nicol DJ, Macfarlane‐Dick D. Formative assessment and self‐regulated learning: A model and seven principles of good feedback practice. Studies in higher education. 2006;31(2):199-218. <https://doi.org/10.1080/03075070600572090>

10. Dijkstra J, Van der Vleuten C, Schuwirth L. A new framework for designing programmes of assessment. Advances in health sciences education. 2010;15(3):379-93. <https://doi.org/10.1007/s10459-009-9205-z>

11. Dijkstra J, Galbraith R, Hodges BD, McAvoy PA, McCrorie P, Southgate LJ, et al. Expert validation of fit-for-purpose guidelines for designing programmes of assessment. BMC medical education. 2012;12(1):20. <https://doi.org/10.1186/1472-6920-12-20>

12. Norcini J, Anderson B, Bollela V, Burch V, Costa MJ, Duvivier R, et al. Criteria for good assessment: consensus statement and recommendations from the Ottawa 2010 Conference. Medical teacher. 2011;33(3):206-14. <https://doi.org/10.3109/0142159X.2011.551559>

13. Cilliers FJ, Schuwirth LWT, Herman N, Adendorff HJ, van der Vleuten CPM. A model of the pre-assessment learning effects of summative assessment in medical education. Advances in Health Sciences Education. 2012;17(1):39-53. <https://doi.org/10.1007/s10459-011-9292-5>

14. ASPIRE. Aspire recognition of excellence in assessment in a medical school. Available on: <http://www.aspire-to-excellence.org/Areas+of+Excellence/.2013>.

15. ASPIRE. ASPIRE International recognition of excellence in education. Available on: <http://www.aspire-to-excellence.org/About+Aspire/2013>.

16. Naveh-Benjamin M, McKeachie WJ, Lin Y-g, Holinger DP. Test anxiety: Deficits in information processing. Journal of Educational Psychology. 1981;73(6):816-24. <https://doi.org/10.1037/0022-0663.73.6.816>

17. Sarason IG. Introduction to the study of test anxiety. In: Sarason IG, editor. Test anxiety: Theory, research, and applications. Hillsdale, NJ: Lawrence Erlbaum Assoc Incorporated; 1980. p. 3-14.

18. Hodapp V, Henneberger A. Test anxiety, study habits, and academic performance. In: Spielberger CD, van der Ploeg HM, Schwarzer R, editors. Advances in test anxiety research. 2. Lisse, the Netherlands: Swets and Zeitlinger; 1983. p. 119-27. (Retrieved from:<https://www.researchgate.net/profile/Wim-Kleijn/publication/15307613_Cognition_Study_Habits_Test_Anxiety_and_Academic_Performance/links/56f06bd008ae70bdd6c94b77/Cognition-Study-Habits-Test-Anxiety-and-Academic-Performance.pdf>).

19. Smith RJ, Arnkoff DB, Wright TL. Test anxiety and academic competence: A comparison of alternative models. Journal of Counseling Psychology. 1990;37(3):313-21. <https://doi.org/10.1037/0022-0167.37.3.313>

20. Carver CS, Scheier MF. Origins and functions of positive and negative affect: A control-process view. Psychological Review. 1990;97(1):19-35. <https://doi.org/10.1037/0033-295X.97.1.19>

21. Covington MV. Making the grade: A self-worth perspective on motivation and school reform. Making the grade: A self-worth perspective on motivation and school reform. 1992:viii, 351-viii, . <https://doi.org/10.1017/CBO9781139173582>

22. Spielberger CD, Vagg PR. Test anxiety: Theory, assessment, and treatment. Washington, DC: Taylor & Francis; 1995.

23. Liberati A, Altman DG, Tetzlaff J, Mulrow C, Gøtzsche PC, Ioannidis JPA, et al. The PRISMA statement for reporting systematic reviews and meta-analyses of studies that evaluate health care interventions: explanation and elaboration. PLoS medicine. 2009;6(7):e1000100. <https://doi.org/10.1016/j.jclinepi.2009.06.006>

24. Rukholm EE, Viverais GA. A multifactorial study of test anxiety and coping responses during a challenge examination. Nurse Education Today. 1993;13(2):91-9. <http://doi.org/10.1016/0260-6917(93)90024-V>

25. Malathi A, Damodaran A. Stress due to exams in medical students - Role of yoga. Indian Journal of Physiology and Pharmacology. 1999;43(2):218-24.

26. Broyles IL, Cyr PR, Korsen N. Open book tests: assessment of academic learning in clerkships. Medical teacher. 2005;27(5):456-62. <http://doi.org/10.1080/01421590500097075>

27. Edelman M, Ficorelli C. A measure of success: Nursing students and test anxiety. Journal for Nurses in Staff Development. 2005;21(2):55-9. <https://doi.org/10.1097/00124645-200503000-00004>

28. Furlong E, Fox P, Lavin M, Collins R. Oncology nursing students' views of a modified OSCE. European journal of oncology nursing : the official journal of European Oncology Nursing Society. 2005;9(4):351-9. <http://doi.org/10.1016/j.ejon.2005.03.001>

29. Sansgiry S, Bhosle M, Dutta AP. Predictiors of test anxiety in doctor of pharmacy students: An empirical study. Pharmacy Education. 2005;5(2):121-9. <http://doi.org/10.1080/15602210500176941>

30. Rohe DE, Barrier PA, Clark MM, Cook DA, et al. The Benefits of Pass-Fail Grading on Stress, Mood, and Group Cohesion in Medical Students. Mayo Clinic Proceedings. 2006;81(11):1443-8. <http://doi.org/10.4065/81.11.1443>

31. Sansgiry, Sail K. Effect of Students' Perceptions of Course Load on Test Anxiety. American Journal of Pharmaceutical Education. 2006;70(2):6-26. <http://doi.org/10.5688/aj700226>

32. Stewart RA, Hauge LS, Stewart RD, Rosen RL, Charnot-Katsikas A, Prinz RA. A CRASH course in procedural skills improves medical students' self-assessment of proficiency, confidence, and anxiety. American journal of surgery. 2007;193(6):771-3. <http://doi.org/10.1016/j.amjsurg.2007.01.019>

33. Hashmat S, Hashmat M, Amanullah F, Aziz S. Factors causing exam anxiety in medical students. J Pak Med Assoc. 2008;58(4):167-70.

34. Pahwa B, Goyal S, Srivastava K, Saldanha D, Bhattacharya D. A study of exam related anxiety amongst medical students. Industrial Psychiatry Journal. 2008;17(1):46-8.

35. Bloodgood RA, Short JG, Jackson JM, Martindale JR. A Change to Pass/Fail Grading in the First Two Years at One Medical School Results in Improved Psychological Well-Being. Academic Medicine. 2009;84(5):655-62. <http://doi.org/10.1097/ACM.0b013e31819f6d78>

36. Brand HS, Schoonheim-Klein M. Is the OSCE more stressful? Examination anxiety and its consequences in different assessment methods in dental education. European journal of dental education : official journal of the Association for Dental Education in Europe. 2009;13(3):147-53. <http://doi.org/10.1111/j.1600-0579.2008.00554.x>

37. Dayalan H, Subramanian S, Elango T. Psychological well-being in medical students during exam stress-influence of short-term practice of mind sound technology. Indian journal of medical sciences. 2010;64(11):501-7. <https://doi.org/10.4103/0019-5359.102122>

38. Spring L, Robillard D, Gehlbach L, Moore Simas TA. Impact of pass/fail grading on medical students' well-being and academic outcomes. Medical Education. 2011;45(9):867-77. <http://doi.org/10.1111/j.1365-2923.2011.03989.x>

39. O'Carroll PJ, Fisher P. Metacognitions, worry and attentional control in predicting OSCE performance test anxiety. Medical education. 2013;47(6):562-8. <http://doi.org/10.1111/medu.12125>

40. Weeks BK, Horan SA. A video-based learning activity is effective for preparing physiotherapy students for practical examinations. Physiotherapy. 2013;99(4):292-7. <https://doi.org/10.1016/j.physio.2013.02.002>

41. Encandela J, Gibson C, Angoff N, Leydon G, Green M. Characteristics of test anxiety among medical students and congruence of strategies to address it. Medical education online. 2014;19:25211. <http://doi.org/10.3402/meo.v19.25211>

42. Johnson CE. Effect of Aromatherapy on Cognitive Test Anxiety Among Nursing Students. Alternative & Complementary Therapies. 2014;20(2):84-7. <http://doi.org/10.1089/act.2014.20207>

43. Lyndon MP, Strom JM, Alyami HM, Yu T-C, Wilson NC, Singh PP, et al. The relationship between academic assessment and psychological distress among medical students: a systematic review. Perspectives on Medical Education. 2014;3(6):405-18. <http://doi.org/10.1007/s40037-014-0148-6>

44. Muldoon K, Biesty L, Smith V. 'I found the OSCE very stressful': student midwives' attitudes towards an objective structured clinical examination (OSCE). Nurse education today. 2014;34(3):468-73. <http://doi.org/10.1016/j.nedt.2013.04.022>

45. Rajiah K, Saravanan C. The effectiveness of psychoeducation and systematic desensitization to reduce test anxiety among first-year pharmacy students. American Journal of Pharmaceutical Education. 2014;78(9). <http://doi.org/10.5688/ajpe789163>

46. Røykenes K, Smith K, Larsen TM. 'It is the situation that makes it difficult': experiences of nursing students faced with a high-stakes drug calculation test. Nurse education in practice. 2014;14(4):350-6. <https://doi.org/10.1016/j.nepr.2014.01.004>

47. Shapiro AL. Test anxiety among nursing students: A systematic review. Teaching and Learning in Nursing. 2014;9(4):193-202. <http://doi.org/10.1016/j.teln.2014.06.001>

48. Young I, Montgomery K, Kearns P, Hayward S, Mellanby E. The benefits of a peer-assisted mock OSCE. Clinical Teacher. 2014;11(3):214-8. <http://doi.org/10.1177/0739986391013100210.1111/tct.12112>

49. Ali M, Asim H, Edhi AI, Hashmi MD, Khan MS, Naz F, et al. Does academic assessment system type affect levels of academic stress in medical students? A cross-sectional study from Pakistan. Medical education online. 2015;20(1):27706. <http://doi.org/10.3402/meo.v20.27706>

50. Chen Y, Henning M, Yielder J, Jones R, Wearn A, Weller J. Progress testing in the medical curriculum: students approaches to learning and perceived stress. BMC Medical Education. 2015;15. <https://doi.org/10.1186/s12909-015-0426-y>

51. March AL, Robinson C. Assessment of High-Stakes Testing, Hopeful Thinking, and Goal Orientation among Baccalaureate Nursing Students. International Journal of Nursing Education Scholarship. 2015;12(1):1-7. <http://doi.org/10.1515/ijnes-2014-0075>

52. Messineo L, Gentile M, Allegra M. Test-enhanced learning: Analysis of an experience with undergraduate nursing students Approaches to teaching and learning. BMC Medical Education. 2015;15(1). <https://doi.org/10.1186/s12909-015-0464-5>

53. Preoteasa CT, Imre M, Preoteasa E. DENTAL STUDENTS' PSYCHOLOGICAL WELL-BEING DURING EXAMINATION PERIOD AND HOLIDAY. Revista medico-chirurgicală̆ a Societă̆ţ̜ii de Medici ş̧i Naturaliş̧ti din Iaş̧i. 2015;119(2):549-56.

54. Schwartz SM, Evans C, Agur AMR. Comparison of physical therapy anatomy performance and anxiety scores in timed and untimed practical tests. Anatomical Sciences Education. 2015;8(6):518-24. <http://doi.org/10.1002/ase.1508>

55. Turan S, Üner S. Preparation for a postgraduate specialty examination by medical students in Turkey: processes and sources of anxiety. Teaching And Learning In Medicine. 2015;27(1):27-36. <https://doi.org/10.1080/10401334.2014.979186>

56. Turner J, Bartlett D, Andiappan M, Cabot L. Students' perceived stress and perception of barriers to effective study: impact on academic performance in examinations. British Dental Journal. 2015;219(9):453-8. <http://doi.org/10.1038/sj.bdj.2015.850>

57. Bovee B. The impact of collaborative testing on test anxiety. Chiropractic Journal of Australia. 2016;44(3):214-21.

58. Cobbett S, Snelgrove-Clarke E. Virtual versus face-to-face clinical simulation in relation to student knowledge, anxiety, and self-confidence in maternal-newborn nursing: A randomized controlled trial. Nurse Education Today. 2016;45:179-84. <http://doi.org/10.1016/j.nedt.2016.08.004>

59. Crego A, Carrillo-Diaz M, Armfield JM, Romero M. Stress and Academic Performance in Dental Students: The Role of Coping Strategies and Examination-Related Self-Efficacy. Journal of dental education. 2016;80(2):165-72. <http://doi.org/10.1002/j.0022-0337.2016.80.2.tb06072.x>

60. Durning SJ, Dong T, Ratcliffe T, Schuwirth L, Artino AR, Boulet JR, et al. Comparing open-book and closed-book examinations: A systematic review. Academic Medicine. 2016;91(4):583-99. <http://doi.org/10.1097/ACM.0000000000000977>

61. Green M, Angoff N, Encandela J. Test anxiety and United States Medical Licensing Examination scores. The clinical teacher. 2016;13(2):142-6. <http://doi.org/10.1111/tct.12386>

62. Kim K-J. Factors associated with medical student test anxiety in objective structured clinical examinations: a preliminary study. International Journal Of Medical Education. 2016;7:424-7. <http://doi.org/10.5116/ijme.5845.caec>

63. Klausenitz C, Hacker H, Hesse T, Kohlmann T, Endlich K, Hahnenkamp K, et al. Auricular Acupuncture for Exam Anxiety in Medical Students-A Randomized Crossover Investigation. Plos One. 2016;11(12):e0168338-e. <https://doi.org/10.1371/journal.pone.0168338>

64. Tagher CG, Robinson EM. Critical Aspects of Stress in a High-Stakes Testing Environment: A Phenomenographical Approach. Journal of Nursing Education. 2016;55(3):160-3. <https://doi.org/10.3928/01484834-20160216-07>

65. Brodersen LD. Interventions for Test Anxiety in Undergraduate Nursing Students: An Integrative Review. Nursing Education Perspectives. 2017;38(3):131-7. <http://doi.org/10.1097/01.NEP.0000000000000142>

66. Fournier KA, Couret J, Ramsay JB, Caulkins JL. Using collaborative two-stage examinations to address test anxiety in a large enrollment gateway course. Anatomical sciences education. 2017;10(5):409-22. <http://doi.org/10.1002/ase.1677>

67. Kalantari M, Zadeh N, Agahi R, Navabi N, Hashemipour M, Nassab A. Measurement of the levels anxiety, self-perception of preparation and expectations for success using an objective structured clinical examination, a written examination, and a preclinical preparation test in Kerman dental students. Journal of Education and Health Promotion. 2017;6. <http://doi.org/10.4103/jehp.jehp_97_15>

68. Khoshhal KI, Khairy GA, Guraya SY, Guraya SS. Exam anxiety in the undergraduate medical students of Taibah University. Medical teacher. 2017;39(sup1):S22-s6. <http://doi.org/10.1080/0142159x.2016.1254749>

69. Liu D, Xu B. Test anxiety: perceptions of American community college nursing students. Empirical Research in Vocational Education and Training. 2017;9(1):1-15. <https://doi.org/10.1186/s40461-017-0048-1>

70. Massey D, Byrne J, Higgins N, Weeks B, Shuker MA, Coyne E, et al. Enhancing OSCE preparedness with video exemplars in undergraduate nursing students. A mixed method study. Nurse education today. 2017;54:56-61. <https://doi.org/10.1016/j.nedt.2017.02.024>

71. Patil SG, Aithala MR. Exam anxiety: Its prevalence and causative factors among Indian medical students. National Journal of Physiology, Pharmacy and Pharmacology. 2017;7(12):1323-,8. <http://doi.org/10.5455/njppp.2017.7.0516113072017>

72. Quinn BL, Peters A. Strategies to Reduce Nursing Student Test Anxiety: A Literature Review. Journal of Nursing Education. 2017;56(3):145-51. <https://doi.org/10.3928/01484834-20170222-05>

73. Rice AH, Vogelweid CM, Kitchel T. The Influence of Crib Sheets on Veterinary Students Exam Performance, Perceived Stress, and Retention of Subject Matter Knowledge. NACTA Journal. 2017;61(1):66-72.

74. Cai W, Pan Y, Chai H, Cui Y, Yan J, Dong W, et al. Attentional bias modification in reducing test anxiety vulnerability: a randomized controlled trial. BMC Psychiatry. 2018;18(1):1-. <http://doi.org/10.1186/s12888-017-1517-6>

75. Custer N. Test Anxiety and Academic Procrastination Among Prelicensure Nursing Students. Nursing Education Perspectives. 2018;39(3):162-3. <http://doi.org/10.1097/01.NEP.0000000000000291>

76. Guraya SY, Guraya SS, Habib F, AlQuiliti KW, Khoshhal KI. Medical students' perception of test anxiety triggered by different assessment modalities. Medical teacher. 2018;40(sup1):S49-s55. <http://doi.org/10.1080/0142159x.2018.1465178>

77. Macauley K, Plummer L, Bemis C, Brock G, Larson C, Spangler J. Prevalence and Predictors of Anxiety in Healthcare Professions Students. Health Professions Education. 2018;4(3):176-85. <http://doi.org/10.1016/j.hpe.2018.01.001>

78. Kolagari S, Modanloo M, Rahmati R, Sabzi Z, Ali Jannati A. The Effect of Computer-based Tests on Nursing StudentsÂ’ Test Anxiety: a Quasi-experimental Study. Acta Informatica Medica. 2018;26(2):115-8. <http://doi.org/10.5455/aim.2018.26.115-118>

79. Vanderoost J, Janssen R, Eggermont J, Callens R, De Laet T. Elimination testing with adapted scoring reduces guessing and anxiety in multiplechoice assessments, but does not increase grade average in comparison with negative marking. PLoS ONE. 2018;13(10). <https://doi.org/10.1371/journal.pone.0203931>

80. Zhang N, Walton DM. Why So Stressed? A Descriptive Thematic Analysis of Physical Therapy Students' Descriptions of Causes of Anxiety during Objective Structured Clinical Exams. Physiotherapy Canada. 2018;70(4):356-62. <https://doi.org/10.3138/ptc.2016-102.e>

81. Al-Sahman LA, Al-Sahman RA, Joseph B, Javali MA. Major Factors Causing Examination Anxiety in Undergraduate Dental Students-A Questionnaire Based Cross-Sectional Study. Annals of Medical and Health Sciences Research. 2019;9(6).

82. Alammari MR, Bukhary DM. Factors contributing to prosthodontic exam anxiety in undergraduate dental students. Advances in medical education and practice. 2019;10:31. <https://doi.org/10.2147/AMEP.S187351>

83. Burton BN, Baxter MF. The effects of the leisure activity of coloring on post-test anxiety in graduate level occupational therapy students. The Open Journal of Occupational Therapy. 2019;7(1):7. <http://doi.org/10.15453/2168-6408.1451>

84. Cipra C, Müller-Hilke B. Testing anxiety in undergraduate medical students and its correlation with different learning approaches. PloS one. 2019;14(3):e0210130. <http://doi.org/10.1371/journal.pone.0210130>

85. Gilavand A, Moezzi M, Gilavand S. Test Anxiety in Dental Students: A Study at the Ahvaz Jundishapur University of Medical Sciences, Iran. Journal of Research in Medical and Dental Science. 2019;7(1):108-13. <http://doi.org/10.5812/erms.85884>

86. Loya NS, Jiwane NN. Exam Anxiety in Professional Medical Students. International Journal of Research & Review. 2019. <https://mail.jpma.org.pk/PdfDownload/1364>

87. Manansingh S, Tatum SL, Elsa-Sofia M. Effects of Relaxation Techniques on Nursing Students' Academic Stress and Test Anxiety. Journal of Nursing Education. 2019;58(9):534-7. <http://doi.org/10.3928/01484834-20190819-07>

88. Michael K, Lyden E, Custer T. Open-Book Examinations (OBEs) in an Ultrasound Physics Course: A Good Idea or a Bad Experiment? Journal of Diagnostic Medical Sonography. 2019;35(3):174-80. <http://doi.org/10.1177/8756479318821075>

89. Poorman SG, Mastorovich ML, Gerwick M. Interventions for test anxiety: How faculty can help. Teaching and Learning in Nursing. 2019;14(3):186-91. <http://doi.org/10.1016/j.teln.2019.02.007>

90. Son HK, So W-Y, Kim M. Effects of Aromatherapy Combined with Music Therapy on Anxiety, Stress, and Fundamental Nursing Skills in Nursing Students: A Randomized Controlled Trial. International journal of environmental research and public health. 2019;16(21). <http://doi.org/10.3390/ijerph16214185>

91. Tsegay L, Shumet S, Damene W, Gebreegziabhier G, Ayano G. Prevalence and determinants of test anxiety among medical students in Addis Ababa Ethiopia. BMC medical education. 2019;19(1):423. <http://doi.org/10.1186/s12909-019-1859-5>

92. Warshawski S, Bar-Lev O, Barnoy S. Role of Academic Self-efficacy and Social Support on Nursing Students' Test Anxiety. Nurse Educator. 2019;44(1):E6-E10. <http://doi.org/10.1097/NNE.0000000000000552>

93. Yusefzadeh H, Iranagh JA, Nabilou B. The effect of study preparation on test anxiety and performance: a quasi-experimental study. Advances in Medical Education and Practice. 2019;10:245. <http://doi.org/10.2147/AMEP.S192053>

94. Zhang N, Henderson CN. Predicting stress and test anxiety among 1st-year chiropractic students. Journal of Chiropractic Education. 2019;33(2):133-9. <http://doi.org/10.7899/JCE-18-11>

95. Akbari V, Asayesh H, Sharififard F, Qorbani M, Elahi A, Heidarpour A. Breakfast consumption as a test anxiety predictor among paramedical students. Journal of Nursing and Midwifery Sciences. 2020;7(1):47. <http://doi.org/10.4103/JNMS.JNMS_37_19>

96. Naeim M, Rezaeisharif A, Zandian H. The Relationship Between Internet Addiction and Social Adjustment, and Test Anxiety of the Students of Ardabil University of Medical Sciences. Shiraz E-Medical Journal. 2020(In Press). <http://doi.org/10.5812/semj.99209>

97. REHMAN F, SAEED I, ZUBAIRI MU. Influencing Factors of Examination Anxiety among Medical Students in Lahore-Pakistan. Pakistan Journal of Medical Sciences. 2020.

98. Mason M. Sample size and saturation in PhD studies using qualitative interviews. Forum qualitative Sozialforschung/Forum: qualitative social research. 2010;11(3). <https://doi.org/10.17169/fqs-11.3.1428>

99. Hennink MM, Kaiser BN, Weber MB. What Influences Saturation? Estimating Sample Sizes in Focus Group Research. Qualitative Health Research. 2019;29(10):1483-96. <https://doi.org/10.1177/1049732318821692>

100. Braun V, Clarke V. Using thematic analysis in psychology. Qualitative Research in Psychology. 2006;3(2):77-101. <https://doi.org/10.1191/1478088706qp063oa>

101. Lynn MR. Determination and quantification of content validity. Nursing Research. 1986;35(6):382-5. <http://doi.org/10.1177/0739986391013100210.1097/00006199-198611000-00017>

102. Wynd CA, Schmidt B, Schaefer MA. Two Quantitative Approaches for Estimating Content Validity. Western Journal of Nursing Research. 2003;25(5):508-18. <https://doi.org/10.1177/0193945903252998>

103. Grant JS, Davis LL. Selection and use of content experts for instrument development. Research in Nursing & Health. 1997;20(3):269-74. <https://doi.org/10.1002/(SICI)1098-240X(199706)20:3><269::AID-NUR9>3.0.CO;2-G

104. Polit DF, Beck CT. The content validity index: are you sure you know what's being reported? Critique and recommendations. Research in nursing & health. 2006;29(5):489-97. <http://doi.org/10.1177/0739986391013100210.1002/nur.20147>

105. Yusoff MSB. ABC of content validation and content validity index calculation. Resource. 2019;11(2):49-54. <https://doi.org/10.21315/eimj2019.11.2.6>

106. Yusoff MSB. ABC of response process validation and face validity index calculation. Education in Medicine Journal. 2019;11(3):55-61. <https://doi.org/10.21315/eimj2019.11.3.6>

107. Cook DA, Beckman TJ. Current Concepts in Validity and Reliability for Psychometric Instruments: Theory and Application. The American Journal of Medicine. 2006;119(2):166.e7-.e16. <https://doi.org/10.1016/j.amjmed.2005.10.036>

108. Mohamad Marzuki MF, Yaacob NA, Yaacob NM. Translation, Cross-Cultural Adaptation, and Validation of the Malay Version of the System Usability Scale Questionnaire for the Assessment of Mobile Apps. JMIR Hum Factors. 2018;5(2):e10308. <https://doi.org/10.2196/10308>
